# Engineering receptor-binding domain and heptad repeat domains towards the development of multi-epitopes oral vaccines against SARS-CoV-2 variants

**DOI:** 10.1371/journal.pone.0306111

**Published:** 2024-08-15

**Authors:** Nur Farhanah Arshad, Fariza Juliana Nordin, Lian Chee Foong, Lionel Lian Aun In, Michelle Yee Mun Teo

**Affiliations:** 1 Department of Biotechnology, Faculty of Applied Sciences, UCSI University, Kuala Lumpur, Malaysia; 2 Department of Biological Sciences and Biotechnology, Faculty of Science & Technology, Universiti Kebangsaan Malaysia, Bangi, Selangor, Malaysia; 3 State Key Laboratory of Systems Medicine for Cancer, Renji-Med X Clinical Stem Cell Research Center, Ren Ji Hospital, School of Medicine, Shanghai Jiao Tong University, Shanghai, China; National Institute of Health, INDIA

## Abstract

The inability of existing vaccines to cope with the mutation rate has highlighted the need for effective preventative strategies for COVID-19. Through the secretion of immunoglobulin A, mucosal delivery of vaccines can effectively stimulate mucosal immunity for better protection against SARS-CoV-2 infection. In this study, various immunoinformatic tools were used to design a multi-epitope oral vaccine against SARS-CoV-2 based on its receptor-binding domain (RBD) and heptad repeat (HR) domains. T and B lymphocyte epitopes were initially predicted from the RBD and HR domains of SARS-CoV-2, and potential antigenic, immunogenic, non-allergenic, and non-toxic epitopes were identified. Epitopes that are highly conserved and have no significant similarity to human proteome were selected. The epitopes were joined with appropriate linkers, and an adjuvant was added to enhance the vaccine efficacy. The vaccine 3D structure constructs were docked with toll-like receptor 4 (TLR-4) and TLR1-TLR2, and the binding affinity was calculated. The designed multi-epitope vaccine construct (MEVC) consisted of 33 antigenic T and B lymphocyte epitopes. The results of molecular dockings and free binding energies confirmed that the MEVC effectively binds to TLR molecules, and the complexes were stable. The results suggested that the designed MEVC is a potentially safe and effective oral vaccine against SARS-CoV-2. This *in silico* study presents a novel approach for creating an oral multi-epitope vaccine against the rapidly evolving SARS-CoV-2 variants. These findings offer valuable insights for developing an effective strategy to combat COVID-19. Further preclinical and clinical studies are required to confirm the efficacy of the MEVC vaccine.

## Introduction

Coronavirus disease 2019 (COVID-19) is an infectious disease caused by the severe acute respiratory syndrome coronavirus 2 (SARS-CoV-2) emerged in Wuhan, China in December 2019. The coronavirus was believed to be transmitted from bats to humans [1–4] which then evolved to adopt a human receptor as its entry mechanism and is spreading until today. The pandemic has resulted in more than 767 million cases and almost 6.9 million total deaths worldwide as of June 2023 [5]. The severity of the disease triggered many research efforts worldwide to quickly develop safe and effective vaccines to fight against the pandemic [6]. It has been shown that the distribution of the first COVID-19 vaccine for emergency use has had a positive impact on bending the curve of COVID-19 as cases and deaths declined remarkably [7, 8].

Although SARS-CoV-2 variants of concerns (VOCs) are no longer circulating and have been de-escalated from the list of the SARS-CoV-2 VOCs as of March 2023, there are still variants of interest (VOIs) emerging from the Omicron variant, XBB.1.5 and XBB.1.16, and it is expected that the SARS-CoV-2 virus will continue mutating and infecting humans [9]. Therefore, efforts in developing a robust and safe COVID-19 vaccines is still ongoing until today. Currently, eleven COVID-19 vaccines have been granted for emergency use by the WHO, with formulations mostly encoding and targeting the surface glycoprotein (S protein) of the SARS-CoV-2, including the use of inactivated whole viruses [10–12]. There were also other 39 COVID-19 vaccines that have been approved by at least one country, including the first intranasal COVID-19 vaccine developed by Bharat Biotech, which is also targeting the S protein of SARS-CoV-2 [11].

Since SARS-CoV-2 is primarily transmitted through human-to-human contact via liquid particles, with infection occurring in the upper respiratory tract [13–15], providing protection on the mucosal surface is one way to prevent viral infection and transmission. Mucosal delivery of vaccines can stimulate mucosal immunity by inducing the secretion of immunoglobulin A (sIgA), which is not effectively induced by vaccines delivered parenterally [15–17]. Currently approved COVID-19 vaccines, including most those under development, are administered via intramuscular injection, and are poorly effective in inducing mucosal immunity [15, 16]. Nevertheless, the newly approved intranasal COVID-19 vaccine, known as BBV154, has shown promising results and significant advantages over existing intramuscular COVID-19 vaccines [18]. It has been shown that the BBV154 vaccine, encoding the wild-type SARS-CoV-2 S protein, successfully stimulated higher production of mucosal antibodies than the intramuscularly injected COVID-19 vaccines [18, 19]. Therefore, many efforts have been devoted to developing more COVID-19 vaccines that can be administered via intranasal or oral routes.

This study focuses on designing a safe and robust multi-epitope oral vaccine against SARS-CoV-2 variants through a reverse vaccinology approach. The computational-based prediction of the potential antigenic epitopes and evaluation of the physicochemical properties of the designed vaccine constructs would help rule out possible unfavourable outcomes that could be caused by the vaccines [20]. Similar methodologies to those used to design previous multi-epitope were adapted in this study to predict highly conserved antigenic and immunogenic epitopes within the SARS-CoV-2 S protein that stimulate T and B-cell activations [21–23]. *In silico* cloning and molecular dockings were also used to evaluate the possibility of the designed vaccine constructs to be efficiently expressed *in vitro* and elicit specific immune responses without allergic or toxic reactions.

## Methodology

### Retrieval of SARS-CoV-2 S protein nucleotide sequences

The nucleotide sequences of the S protein for SARS-CoV-2 Wuhan-isolate (reference sequence) and the previous five variants of concerns (VOCs): Omicron (B.1.1.529), Alpha (B.1.1.7), Beta (B.1.351), Gamma (P.1), and Delta (B.1.617.2) were obtained in FASTA format from the NCBI database (https://ncbi.nlm.nih.gov). Data were collected between January 2021 to 2023 from each region available in the NCBI database–Asia, Africa, North America, South America, Europe and Oceania. Only complete nucleotide sequences with no ambiguous characters (unknown bases) were selected in our study. The nucleotide or protein sequences of the plasmid vector pNZ8149 and the defensin (adjuvant), were also retrieved in FASTA format from the MoBiTec (http://www.mobitec.com) and NCBI databases, respectively. The processed nucleotide acid sequences were aligned using Geneious Prime 2023.0.4 software using MUSCLE alignment, and translated using Expasy online server (https://web.expasy.org/translate/) to obtain the protein sequences for further analysis. The regions of interest from the S protein of SARS-CoV-2 for the design of the multi-epitope vaccine construct, were extracted based on the location of the domains obtained from the Conserved Domain Database (CDD) web server (https://www.ncbi.nlm.nih.gov/Structure/cdd/wrpsb.cgi). Specifically, the RBD domain (RBD: 319–541), and the region from the HR1 to HR2 domain of the S2 subunit (HRs: 918–1203) were extracted.

### Prediction of critical binding epitopes

#### Cytotoxic T-cell epitopes

**Fig 1:**
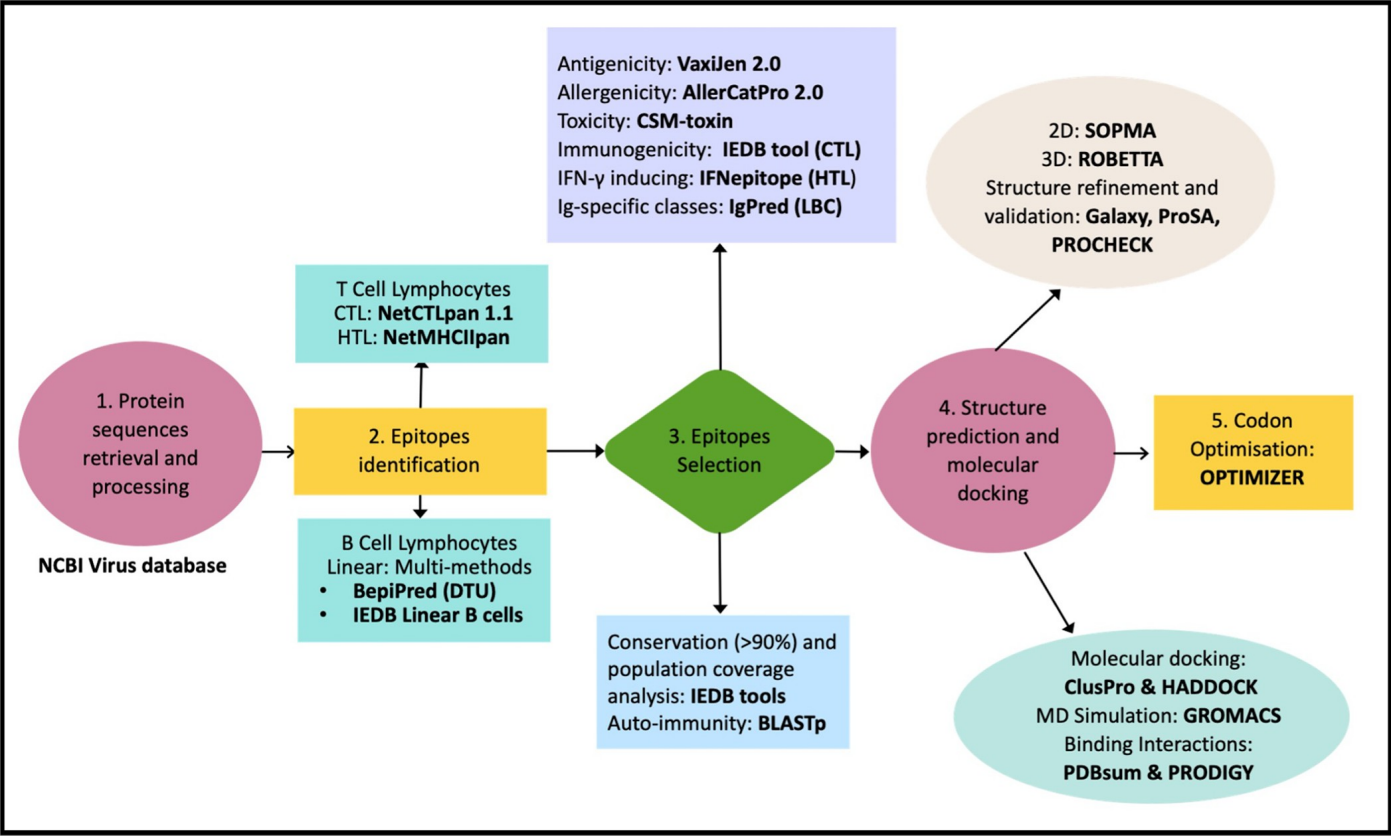
Flowchart summarising the immunoinformatic methods in the rational design of the vaccine. The formation of vaccine construct *in silico* includes 1. Protein sequences retrieval and processing, 2. Epitopes identification, 3. Epitopes selection, 4. Structure prediction and molecular docking and 5. Codon optimisation. Tools and web servers used in the analyses are in bold. CTL: Cytotoxic T Lymphocytes; HTL: Helper T Lymphocytes; LBC: Linear B-cells; Ig: immunoglobulin; 2D: secondary structure; 3D: tertiary structure.

The epitopes identification step as described in Fig 1 includes the prediction of the potential cytotoxic T lymphocyte (CTL) epitopes using NetCTLpan v1.1 (https://services.healthtech.dtu.dk/services/NetCTLpan-1.1/) from the DTU server. This method is optimised and trained to predict CTL epitopes with high specificity, outperforming other CTL epitope predictors [24]. The tool predicts the 9-mer peptides that act as the MHC ligands interacting with the 27 most frequent HLA class I supertypes in the global population, including HLA-A*01:01, HLA-A*02:01, HLA-A*02:03, HLA-A*02:06, HLA- A*03:01, HLA-A*11:01, HLA-A*23:01, HLA-A*24:02, HLA-A*26:01, HLA- A*30:01, HLA-A*30:02, HLA-A*31:01, HLA-A*32:01, HLA-A*33:01, HLA- A*68:01, HLA-A*68:02, HLA-B*07:02, HLA-B*08:01, HLA-B*15:01, HLA- B*35:01, HLA-B*40:01, HLA-B*44:02, HLA-B*44:03, HLA-B*51:01, HLA- B*53:01, HLA-B*57:01, HLA-B*58:01 [25]. The threshold parameters were set to the default values. Predicted epitopes with high binding affinity to the selected HLA class supertype will have a score close to 1 in the output results [26].

#### Helper T-cell epitopes

Helper T-cells (Th) are important members involved in the adaptive immunity that recognise foreign antigens and help to stimulate the activation of other immune cells [27]. Therefore, the identification of antigenic epitopes that can be recognised by Th cells are important to stimulate the adaptive immunity. The helper T lymphocyte (HTL) epitopes were predicted using the NetMHCIIpan v4.1 from the DTU server (https://services.healthtech.dtu.dk/services/NetMHCIIpan-4.0/). The length of the predicted epitopes for MHC class II was set to a default of 15-mer and the threshold for identifying strong and weak binders were set to the values of 1 and 5, respectively. A set of 27 HLA class II supertypes (HLA-DRB1*01:01, HLA-DRB1*03:01, HLA- DRB1*04:01, HLA-DRB1*04:05, HLA-DRB1*07:01, HLA-DRB1*08:02, HLA- DRB1*09:01, HLA-DRB1*11:01, HLA-DRB1*12:01, HLA-DRB1*13:02, HLA- DRB1*15:01, HLA-DRB3*01:01, HLA-DRB3*02:02, HLA-DRB4*01:01, HLA- DRB5*01:01, HLA-DQA1*05:01/DQB1*02:01, HLA-DQA1*05:01/DQB1*03:01, HLA-DQA1*03:01/DQB1*03:02, HLA-DQA1*01:01/DQB1*05:01, HLA-DPA1*02:01/DPB1*01:01, HLA-DPA1*01:03/DPB1*04:01, HLA-DPA1*02:01/DPB1*05:01, HLA-DPA1*02:01/DPB1*14:01) that were most frequent in the global population were selected for the prediction of the HTL epitopes. The programme gives an output of the predicted peptides along with the complimentary alleles and predicted binding scores between the peptides and the alleles.

#### Linear B-cell epitopes

B-cell epitopes are antigenic fragments that can be recognised by antibodies and are important components in triggering humoral immunity [28]. In this study, we used multi-method approach to predict the linear B-cell epitopes (LBC) from the amino acid sequences of the SARS-CoV-2 S protein. The prediction was performed using BepiPred v3.0 (https://services.healthtech.dtu.dk/services/BepiPred-3.0/), as well as IEDB Antibody Epitope Prediction tool (http://tools.iedb.org/bcell/) for Chou & Fasman Beta-Turn, Emini Surface Accessibility prediction, Karplus & Schulz Flexibility, Kolaskar & Tongaonkar, and Parker Hydrophilicity. The candidate LBC epitopes were shortlisted to those that were between 12-16-mer, antigenic, non-allergenic, non-toxic, and potentially capable to induce either IgG or IgA antibodies.

The Ig-class specific antibodies for the candidate LBC epitopes were predicted using the Variable Length Model of the IgPred tool (https://webs.iiitd.edu.in/raghava/igpred/index.html). The predicted LBC epitopes were evaluated for their ability to induce IgA, IgG and IgE antibodies with approximately 80% accuracy. The threshold for each antibody-specific class was set to 0.0 for a balanced confidence and sensitivity prediction of the B-cell epitopes specific antibodies. The LBC epitopes predicted to induce IgE antibody were removed to avoid triggering any allergic reactions.

The shortlisted LBC epitopes were validated against the IEDB database (https://iedb.org/home_v3.php) and the predicted LBC epitopes that have been reported and identified by positive B-cell assays were shortlisted.

### Epitopes selection

Following epitopes identification step, the epitopes were filtered out based on their antigenicity, allergenicity, toxicity, immunogenicity, conservancy and auto-immune responses (Fig 1).

#### Antigenicity, allergenicity and toxicity analyses

To test the antigenicity, allergenicity and toxicity of the shortlisted epitopes, VaxiJen v2.0 (http://www.ddg-pharmfac.net/vaxijen/VaxiJen/VaxiJen.html), AllerCatPro v2.0 server (https://allercatpro.bii.a-star.edu.sg), and CSM-toxin (https://biosig.lab.uq.edu.au/csm_toxin/) web servers, were used. The antigenicity threshold for the epitopes was set to 0.4, with ‘Virus’ set as the target organism. Epitope and whole vaccine constructs with antigenicity value higher than 0.4 were recognised as probable antigens. The entire vaccine constructs formulated after the *in silico* study were also assessed using similar methods and tools.

#### Prediction of immunogenicity and IFN-γ inducing epitopes

The immunogenicity score of each potential CTL epitope was calculated using the IEDB Class I Immunogenicity tool (http://tools.iedb.org/immunogenicity/), to filter only the epitopes that can stimulate cytotoxic lymphocytes responses for our vaccine design. Peptides with positive scores were selected for further analysis. The predicted HTL epitopes were evaluated using IFNepitope web server (http://crdd.osdd.net/raghava/ifnepitope/predict.php), and epitopes that were predicted to bind with IFN-γ, which is significant in inducing anti-viral activity, were selected [29].

#### Conservancy and population coverage analysis

The percentage conservancy of the predicted epitopes in SARS-CoV-2 S protein was checked using the IEDB Epitope Conservancy Analysis web server (http://tools.iedb.org/conservancy/). To design a mutational-resistant multi-epitope vaccine against SARS-CoV-2, epitopes that were highly conserved in the SARS-CoV-2 variants where the epitopes were present in more than 90% of the protein sequences retrieved, were selected. Since the HLA genotype frequencies also vary across different human populations and will influence the binding affinities between the vaccine and HLA I and II molecules [30], population coverage analysis was performed on the multi-epitope vaccine construct (MEVC) using the IEDB population coverage calculation tool (http://tools.iedb.org/population/) [30] on the selected epitopes and their corresponding HLA alleles [21].

#### Sequence homology and comparison to human proteome

The selected epitopes were compared for human proteome homologs using NCBI Protein BLAST (BLASTp) (https://blast.ncbi.nlm.nih.gov/Blast.cgi?PAGE=Proteins) to prevent stimulation of autoimmune response in the host. The analysis was restricted to proteins from *H*. *sapiens* (taxid:9606) with E-value threshold between 0.01 to 0.0001 [31]. Peptides that have non-significant similarity to human proteome were chosen and included in the vaccine construct.

### *In silico* vaccine construction

**Fig 2:**
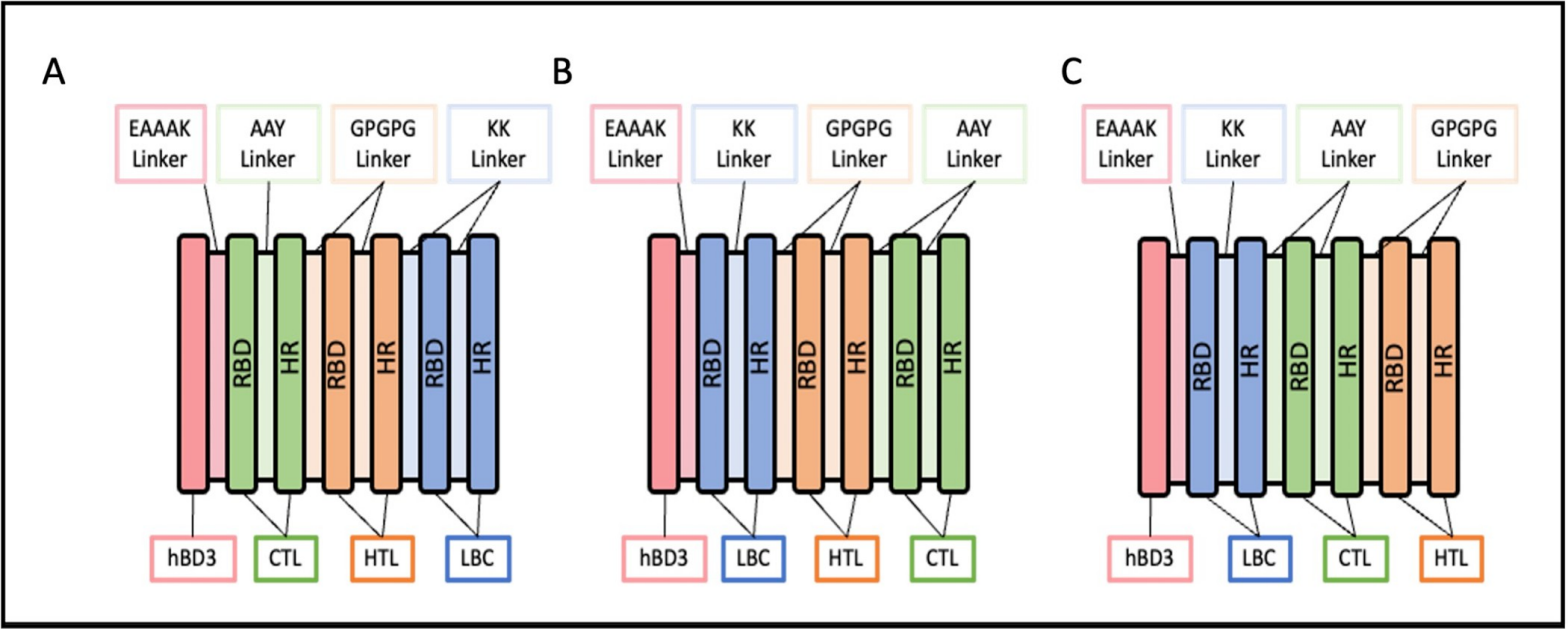
Three proposed organisation (A, B and C) of the multi-epitope vaccine constructs, MEVC-A, MEVC-B and MEVC-C. The orientation of the epitopes made based on previous literatures [26, 33, 34] were tested for the efficacy of the vaccine *in silico*. The defensin adjuvant (pink) was joined to the epitopes by the EAAAK linker (light pink). CTL epitopes (green) were joined together with AAY linker (light green). The HTL epitopes (orange) were separated with GPGPG linker (light orange) while LBC epitope (blue) were joined with KK linkers (light blue). The epitopes were then arranged from the most conserved epitopes to the least conserved extracted from the receptor-binding domain (RBD) S1 subunit and S2 (HR) subunits. hBD3, human β-defensin 3 (adjuvant); CTL, Cytotoxic Lymphocytes; HTL, Helper T Lymphocytes; LBC, Linear B-cells [26, 33, 34].

To construct the multi-epitope vaccine construct (MEVC), the finalised epitopes from previous steps (Epitopes Selection) were used, and the correct orientation of the fusion domains were employed for efficient *in vitro* and *in vivo* expression. T and B-cell epitopes were arranged together in the MEVC, separated by commonly used linkers (Fig 2). The KK and GPGPG linkers were used to separate the HTL and B-cell epitopes, respectively. While AAY linker was used to separate CTL epitopes, as this linker can act as the cleavage site for the proteasome to process the CTL suitable for the TAP transporter [32].

The adjuvant, human β-defensin 3 (hBD3), was also included in the MEVC, separated by EAAAK linker from the epitopes at the N-terminal of the MEVC to enhance the efficacy of the vaccine [35–42]. To assist in the protein purification process in further *in vitro* study, a 6×His-tag was included at the C-terminal of the MEVCs linked with RVRR linker. A signal peptide comprised of 27 amino acids, Usp45, was also added upstream to the adjuvant to allow the secretion of the heterologous recombinant protein from the *L*. *lactis*.

#### Prediction of physicochemical properties of vaccine construct

The Expasy ProtParam online server (https://web.expasy.org/protparam/) was used to analyse the physicochemical properties of the MEVCs, including molecular weight, theoretical isoelectric point (pI), amino acid distributions, amino acid composition, estimated half-life, instability index, aliphatic index, and the grand average of hydropathicity (GRAVY) [21, 26]. The theoretical pI determines the acidic or basic nature of the vaccine. The instability index of the protein that is lesser than 40 is considered stable [21] while the estimated aliphatic index, which represents the thermostable nature of the protein, is indicated as thermo-stable when the value is between 61.09 to 83.59 [43]. A negative value of the GRAVY represents the hydrophilic nature of the input subunit vaccine [43]. The MEVCs that were stable and had high estimated half-life were selected for further analysis.

The solubility of the vaccine constructs was predicted using Protein-sol web server (https://protein-sol.manchester.ac.uk/cgi-bin/solubility/sequenceprediction.php) [44], which compares the property of the amino acid composition of the input query amino acid sequences with an experimental dataset of *E*. *coli* soluble proteins [44]. A scaled solubility value of more than 0.45 was interpreted to have higher solubility than average soluble *E*. *coli* proteins [44, 45].

#### Secondary and 3D structure analyses

The secondary structures of the MEVCs were predicted using SOPMA (https://npsa-prabi.ibcp.fr/cgi-bin/npsa_automat.pl?page=/NPSA/npsa_sopma.html) while the 3D structures of the MEVCs were modelled using RoseTTAFold modelling method in the Robetta web server. The RoseTTAfold method is an improved deep learning modelling method which can accurately model proteins with no known homologs and outperformed other modelling methods provided in the server. The modelling server generated five 3D structures of the input protein sequences based on the modelling method chosen by users.

The 3D structures of the individual epitope selected were also predicted using PEPFOLD3 web server (https://mobyle.rpbs.univ-paris-diderot.fr/cgi-bin/portal.py#forms::PEP-FOLD3), prior to subsequent molecular dockings and binding affinity analyses.

#### Refinement and validation of 3D structure

The 3D structures generated by Robetta server were submitted to the ProSA-web server (https://prosa.services.came.sbg.ac.at/prosa.php) [46] and further validation was performed using PROCHECK web server (https://www.ebi.ac.uk/thornton-srv/software/PROCHECK/) [47]. The ProSA-web calculated a Z-score for the refined 3D structure to define the overall model quality. The model structure with the best quality was refined using GalaxyRefine web server, which output another five model structures that were refined, along with the information on their structural changes in terms of Rama-favoured region, GDT-HA, RMSD and MolProbity score. The GDT-HA score, a high accuracy version of the global distance test–total score (GDT-TS), indicates the structure similarity between the input and experimental structures [48, 49]. A higher GDT-HA score suggests improved model structures [48]. The RMSD score evaluates the changes in structural stability of the refined model structures. The MolProbity scores assess the overall quality of atoms in the input model structure, where lower score indicates better quality of the refined structure [49]. A good quality of model structure should have more than 90% residues within the Rama-favoured region [50]. The models from the GalaxyRefine were re-validated using ProSA and PROCHECK servers, and the model structure with the best quality score was used as our final MEVC structure for docking and *in silico* cloning [21].

#### Molecular docking with TLR4 and TLR1-TLR2 molecules

Molecular dockings were performed between the MEVC with the TLR molecules using ClusPro 2.0 (https://cluspro.bu.edu/) and HADDOCK version 2.4 web servers (https://bianca.science.uu.nl/haddock2.4/) [51–54], as these docking tools have been highly cited in many publications [55–57] and were also ranked as the best tools for protein docking [58]. TLRs can stimulate the activation of antigen-presenting cells (APCs) and contribute to the effectiveness of the vaccine [59]. The protein-protein complex stability score were considered in this analysis. The TLR structure, including TLR4 homodimer (PDB:3FXI) and TLR1-TLR2 heterodimer (PDB:2Z7X), together with the predicted MEVC 3D structure were used as the input. The ClusPro server ranked the generated complexes based on energy score of the clusters [56]. The cluster with the most number of members and most negative energy scores was selected. The CTL and HTL epitopes were also docked with their corresponding HLA alleles and also with the TLR molecules. Owing to the blind docking feature available on the ClusPro web server, we performed the docking on our protein-protein complex without restricting any active sites or interface residues for the binding interaction between the complex due to lack of knowledge on the binding pockets for the MEVC.

To cross-verify the docking results from the ClusPro, we performed another molecular docking procedure on the vaccine construct with the TLR molecules using HADDOCK, and compare the outcomes. However, since HADDOCK is an information-driven docking web server, we were required to input the active residues of the proteins to perform the docking. Therefore, we utilised the information on the interacting residues obtained from previous literature reviews on the residues within the RBD that were identified to be the critical binding epitopes that are interacting with the ACE2 receptor, and the residues within the HR domains interacting with the neutralising antibodies of SARS-CoV-2 [60–62]. We employed the residues between 438–506 within the RBD of the SARS-CoV-2 which has been identified as the receptor binding domain (RBM) [62] and also residues between 1141–1161 from the S2 subunit identified as the spike-specific CD4+ T cell epitopes by Guo *et al*. [61] but only the residues that were predicted to have a relative solvent accessibility confidence higher than five by SABLE web server [63–66] were selected as the active residues for the molecular docking. The docked complexes with the lowest HADDOCK score and restraints violation energy were analysed as the low scores indicated that the docked protein-protein complex were the best complex model generated by HADDOCK.

#### Molecular dynamic (MD) simulations of the docked complexes

Molecular dynamic simulations were conducted on the docked protein-protein complex TLR molecules and the MEVC 3D output generated from the ClusPro web server to assess the stability, flexibility and binding energy of the complexes [67, 68]. The simulations were performed for at least 100ns by employing the all-atom optimized potentials for liquid simulations (OPLS-AA) force field using the GROMACS 2023.4 software. The simulations of the docked complexes were done in a cubic box with a dimension of 1.0nm with spc216, which is a generic equilibrated 3-point water model [69, 70], The net charge on the protein in the simulation system was neutralised by adding a specific concentration of negative ions to perform the energy minimisation and subsequent dynamic simulations. The potential energy of the protein-protein complexes was accessed and the graph plot was visualised using the XMGRACE tool to determine if the energy minimisation was successful [69, 71]. To ensure that the solvent and ions added around the protein were well-equilibrated for the dynamic simulations, the equilibration step was conducted in two phases, NVT (constant Number of particle, Volume, and Temperature) and NPT (Pressure), where the system were equilibrated to achieve a stable experimental conditions for the simulation of the complexes. The target value for the temperature is 300K and for pressure was set to 1 bar. The Root Mean Square Deviation (RMSD) and Root Mean Square Fluctuation (RMSF) of the carbon atom of each chain were calculated from the simulations to predict the structural stability and flexibility of the complexes. The radius of gyration of the protein-protein complex was also assessed to measure the compactness of the complexes. The graphs of the analyses were also plotted and visualised using the XMGRACE plotting tool [71].

#### Protein-protein interaction analysis

The binding interactions between the protein-protein complexes were assessed using PDBsum online web server (http://www.ebi.ac.uk/thornton-srv/databases/pdbsum/Generate.html) [72], with the PDB structure downloaded from ClusPro as the input. The interacting residues between the complexes were identified, and the binding interactions were visualised. The leucine-rich repeats (LRRs) motif of TLR4 molecule were retrieved from the Uniprot server, as this region is important in recognising pathogen-associated molecular patterns (PAMP) [73]. The activation of TLR4 requires a protein molecule known as myeloid differentiation-2 (MD-2), which interacts with the extracellular portion of the TLR4 molecule and promotes TLR4 dimerization [74]. Binding of the MEVC within the LRRs motif of TLR4 and MD-2 can potentially trigger the desired immune responses. In addition, the ligand binding domains of the heterodimer TLR1-TLR2 molecule were retrieved from previous study by Jin *et al*. [75]. It was reported that ligands binding to the residues of the TLR1 (258–339) and TLR2 (266–355) promote the heterodimerisation of TLR1-TLR2 and intracellular TIR domain for subsequent downstream signalling [75].

The binding affinity of the protein complexes were also assessed using PRODIGY web server (https://wenmr.science.uu.nl/prodigy/). The PDB structure of the protein-protein complex generated by ClusPro and HADDOCK were used as input. The binding affinity was calculated based on the interface binding contacts between the MEVC and TLR molecules combined with the properties of the non-interacting residues in the protein-protein complexes [54, 76, 77]. The protein-ligand method, an extension method provided in the PRODIGY web server to predict the binding affinity between small ligand and protein complex [78], was used to evaluate the binding interactions of the candidate epitopes with the HLA alleles [79, 80], where epitopes with stronger binding were selected for further analysis.

#### Codon optimisation

The MEVCs’ protein sequences were reverse translated into DNA sequences using a reverse translation tool from the Sequence Manipulation Suite online server (https://www.bioinformatics.org/sms2/rev_trans.html), based on *L*. *lactis subsp*. *Cremoris* MG1363 codon usage table obtained from the Codon Usage Database (http://www.kazusa.or.jp/codon/). The resulting DNA sequences were further optimised to *L*. *lactis subsp*. *Cremoris* MG1363 genome using the OPTIMIZER web server (http://genomes.urv.es/OPTIMIZER/), to ensure efficient heterologous gene expression *in vivo*, as *L*. *lactis* NZ3900 is the derivative of the *L*. *lactis subsp*. *cremoris* MG1363 [81]. The GC content and Codon Adaptive Index (CAI) values of the DNA sequences were evaluated. GC content between 30% and 70% and a CAI value of more than 0.8 indicates an optimal codon usage with high expression of the gene is expected [26, 43, 69].

## Results

### Retrieval of protein sequences

A total of 78,182 complete nucleotide sequences of SARS-CoV-2 variants (VOCs)—Alpha (B.1.1.7), Beta (B.1.351), Delta (B.1.617.2), Gamma (P.1), Omicron (B.1.1.529)–including the Wuhan reference sequence (NC_045512.2) were extracted and filtered from the NCBI database. Multiple sequence alignment (MSA) of the unique nucleotide sequences of the SARS-CoV-2 virus was performed and trimmed between positions 21,000 to 26,000 to extract the region coding for the S protein of the virus. The nucleotide sequences were translated into amino acids to extract the receptor-binding domain (RBD) and the heptad repeats (HRs) region of the virus. A total of 669 and 718 amino acid sequences coding for the RBD and HRs, respectively, were used for epitope predictions.

### Prediction and selection of the critical binding epitopes

#### T-lymphocyte epitopes

The T-lymphocyte epitopes were divided into two types, Cytotoxic T-lymphocyte (CTL) and Helper T-Lymphocyte (HTL) epitopes, which induce two major cellular immune responses against the SARS-CoV-2 virus. A pool of 9-mer peptides was predicted for the CTL epitopes, resulting in 533 and 361 potential CTL epitopes in the targeted region of the S proteins, RBD and HRs, respectively. Similarly, a total of 1458 and 1245 potential 15-mer HTL epitopes were predicted within the S1 RBD and the S2 HRs region of SARS-CoV-2, respectively. After initial filtration, 267 out of 894 predicted CTL epitopes passed the first filtration process, while 1357 out of 1458 and 1114 out of 1245 predicted HTL epitopes in the RBD and HRs, respectively, passed the first filtration process. The initial filtration of the epitopes indicated that the CTL and HTL epitopes were predicted to be antigenic and immunogenic and can be potentially included in the formation of the vaccine constructs. The antigenicity and immunogenicity filtration stage showed that 627 CTL epitopes and 231 HTL epitopes that were predicted from the NetCTLpan v1.1, and NetMHCIIpan v4.1, respectively, may not have any potential to trigger cellular immune response mediated by the T lymphocytes, and were removed from our candidate lists.

The filtered CTL and HTL epitopes were also confirmed that they were non-allergenic and non-toxic after the second filtration stage. Then, the predicted T-cell epitopes were selected based on the complementary HLA alleles binding to each epitope. Epitopes with overlapping HLA binding alleles were removed. For the CTL epitopes, the ^1060^VVFLHVTYV^1068^, ^1062^FLHVTYVPA^1070^ and ^1192^NLNESLIDL^1200^ were removed as they were predicted to bind to similar HLA I alleles (HLA-A*02:03) as ^1060^VVFLHVTYV^1068^ (S1 Table). The latter epitope was kept as it binds to more different HLA I alleles (HLA-A*02:01, HLA-A*02:03, HLA-A*02:06, HLA-A*68:02) compared to the other two epitopes. In contrast, the ^1016^AEIRASANL^1024^ and ^1181^KEIDRLNEV^1189^ epitopes were predicted to bind to exactly similar HLA I alleles (S1 Table). The ^1181^KEIDRLNEV^118^ epitope was kept as it has higher binding affinity towards the corresponding HLA I alleles compared to ^1016^AEIRASANL^1024^ (not shown). From the HTL epitopes, epitopes with a similar binding core but different alleles binding were selected as the candidate HTL epitopes (S2 Table).

#### Linear B-cell epitopes

The linear B-cell (LBC) epitopes predicted using the multi-method approach resulted in a large pool of candidates. The LBC epitopes that matched the selection criteria which were 12-16-mer in length, antigenic, non-allergenic, and non-toxic, reduced the number of LBC candidates. Within the RBD region of the S protein, eight LBC epitopes were shortlisted, while there were 28 LBC epitopes predicted within the HR1-HR2 region (S3 Table). Of the 36 shortlisted LBC epitopes, 33 of them have the potential ability to induce either IgG or IgA antibodies predicted by the IgPred server at a threshold set to 0.0 (S3 Table).

The LBC candidates were then validated using the database of experimented B-cell epitopes from the IEDB resource. From the comparative analysis between the 36 shortlisted LBC epitopes and the existing linear B-cell epitopes from the IEDB database, the remaining three epitopes that were not predicted to have the ability to induce either IgA and IgG were consistent with the reported LBC from IEDB except ^1067^YVPAQEKNFTTA^1078^, where this epitope was experimentally tested to positively bind to IgM (IEDB_ID: 15908232) and IgG antibodies (IEDB_ID: 15908231). In addition, eight epitopes that were predicted to induce IgG response and four epitopes to induce IgA antibody by the IgPred tool were not experimentally validated (S3 Table). Therefore, another twelve LBC epitopes were removed from our LBC epitope list. The remaining 21 LBC epitope candidate which include eight candidates within the RBD, and thirteen epitopes within HR1-HR2, have the potential to be included in the formation of the vaccine construct.

#### Non-homology peptides to human proteome

The BLASTp results showed that all the candidate T and B lymphocyte epitopes have no significant similarity to the human proteome and were expected to not trigger any autoimmune response in the host.

#### Highly conserved epitopes with broad population coverage

The shortlisted CTL, HTL and LBC epitopes that matched exactly to more than 90% of the fraction of the SARS-CoV-2 S protein sequences retrieved, were selected, as the highly conserved epitopes. A total of nine CTL (Table 1), five HTL epitopes (Table 2), and nineteen LBC epitopes (S4 Table) met the criteria and were finalised for the formation of the multi-epitope vaccine construct (MEVC) *in silico*.

**Table 1:** Final CTL epitope candidate within receptor-binding domain and heptad repeat domains of the SARS-CoV-2 S protein with their antigenicity value and conservancy percentage.

No	Epitope	Region	Antigenicity	Conservancy
1	^388^NDLCFTNVY^396^	RBD	1.6828	98.36%
2	^507^PYRVVVLSF^515^	RBD	1.0281	98.51%
3	^509^RVVVLSFEL^517^	RBD	1.1918	97.91%
4	^349^SVYAWNRKR^357^	RBD	0.7650	96.86%
5	^1059^GVVFLHVTY^1067^	HR	1.4104	98.05%
6	^1060^VVFLHVTYV^1068^	HR	1.5122	97.77%
7	^1181^KEIDRLNEV^1189^	HR	0.5300	95.54%
8	^1065^VTYVPAQEK^1073^	HR	0.8132	94.15%
9	^1113^QIITTDNTF^1121^	HR	0.4253	90.57%

RBD: Receptor-binding domain; HR: Heptad repeat

**Table 2:** Final HTL epitope candidates within receptor-binding domain and heptad repeat domains of the SARS-CoV-2 S protein with their binding core, antigenicity value and conservancy percentage.

No	Epitope	Core	Region	Antigenicity	Conservancy
1	^509^RVVVLSFELLHAPAT^523^	FELLHAPAT	RBD	0.7485	94.17%
2	^431^GCVIAWNSNNLDSKV^445^	IAWNSNNLD	RBD	0.4585	93.57%
3	^1057^PHGVVFLHVTYVPAQ^1071^	VVFLHVTYV	HR	0.8097	95.68%
4	^1059^GVVFLHVTYVPAQEK^1073^	FLHVTYVPA	HR	1.1043	93.45%
5	^1062^FLHVTYVPAQEKNFT^1076^	VTYVPAQEK	HR	1.1908	93.04%

RBD: Receptor-binding domain; HR: Heptad repeat

In the population coverage analysis, the CTL and HTL epitopes were able to cover around 91.61% and 99.83% of the global population, respectively. Since both CTL and HTL epitopes were included in the MEVC, we focused on the combined population coverage of the MHC class I and MHC class II restricted T-cell epitopes [30]. The average global population coverage by class I and class II combined for the final epitope candidates is 99.99% (Table 3).

**Table 3:** Population coverage analysis using IEDB resource tool calculated based on the set of 19 T lymphocytes epitopes selected for the formulation of the vaccine construct globally and specific to geographical regions where the SARS-CoV-2 strains were retrieved.

Population/area	Coverage		
	CTL epitopes (class I)	HTL epitopes (class II)	Class combined
World	91.61%	99.83%	99.99%
Asia	86.15%	98.40%	99.81%
Africa	76.37%	85.89%	96.85%
Europe	93.53%	100.00%	100.00%
North America	93.48%	100.00%	100.00%
South America	81.54%	100.00%	100.00%
Oceania	89.5%	99.97%	100.00%

CTL: Cytotoxic T Lymphocytes; HTL: Helper T Lymphocyte

The broad population coverage predicted for our MEVC ensured the effectiveness of the vaccine for majority of human populations. The population coverage analysis was also performed individually for the six regions where we initially retrieved the SARS-CoV-2 nucleotide sequences–Asia, Africa, North America, South America, Europe and Oceania. The combined population coverage for the six regions is more than 96%, and the maximum coverage (100%), was expected in the four regions i.e., North America, South America, Europe, and Oceania.

#### Validation on the finalised epitopes with IEDB database

To validate the selected epitopes for the formation of the vaccine constructs, each of the CTL, HTL and LBC epitope were cross-checked with the experimental proven epitopes within the IEDB database. Out of the 13 finalised CTL epitopes that we have selected, only three of them, ^388^NDLCFTNVY^396^, ^342^FNATRFASV^350^ and ^1113^QIITTDNTF^1121^, were not experimentally validated with any MHC ligand binding assay or have not been reported to interact with any HLA class I alleles.

From the final candidates of HTL epitopes that were selected, two of eleven epitopes, ^510^VVVLSFELLHAPATV^524^ and ^1059^GVVFLHVTYVPAQEK^1073^, have not been experimentally validated and not reported in the IEDB database. The remaining nine HTL epitopes that were finalised were reported to either have the ability to induce the release of IFN-γ or were interacting with HLA class II alleles in MHC ligand binding assays.

Despite not being reported in the IEDB database and validated by any experimental assays, we still included the three CTL and two HTL epitopes in our vaccine construct formation as these epitopes have shown high potential ability to induce T lymphocyte responses from the *in silico* analyses.

#### Formation of multi-epitope vaccine construct *in silico*

Three different arrangements of the MEVCs (Fig 2A–2C) based on previous literatures were evaluated for antigenicity, toxicity, allergenicity and immunogenicity. All three arrangements of the MEVCs were predicted to be probable antigens, and two MEVC arrangements with high antigenic score, MEVC-B (0.7081) and MEVC-C (0.7104), were selected for further analysis. The MEVCs were also predicted to not stimulate any allergic reactions and were non-toxic, indicating that they were safe to be consumed and have high potential to trigger immune responses.

#### Prediction of physicochemical properties and solubility of vaccine constructs

**Fig 3:**
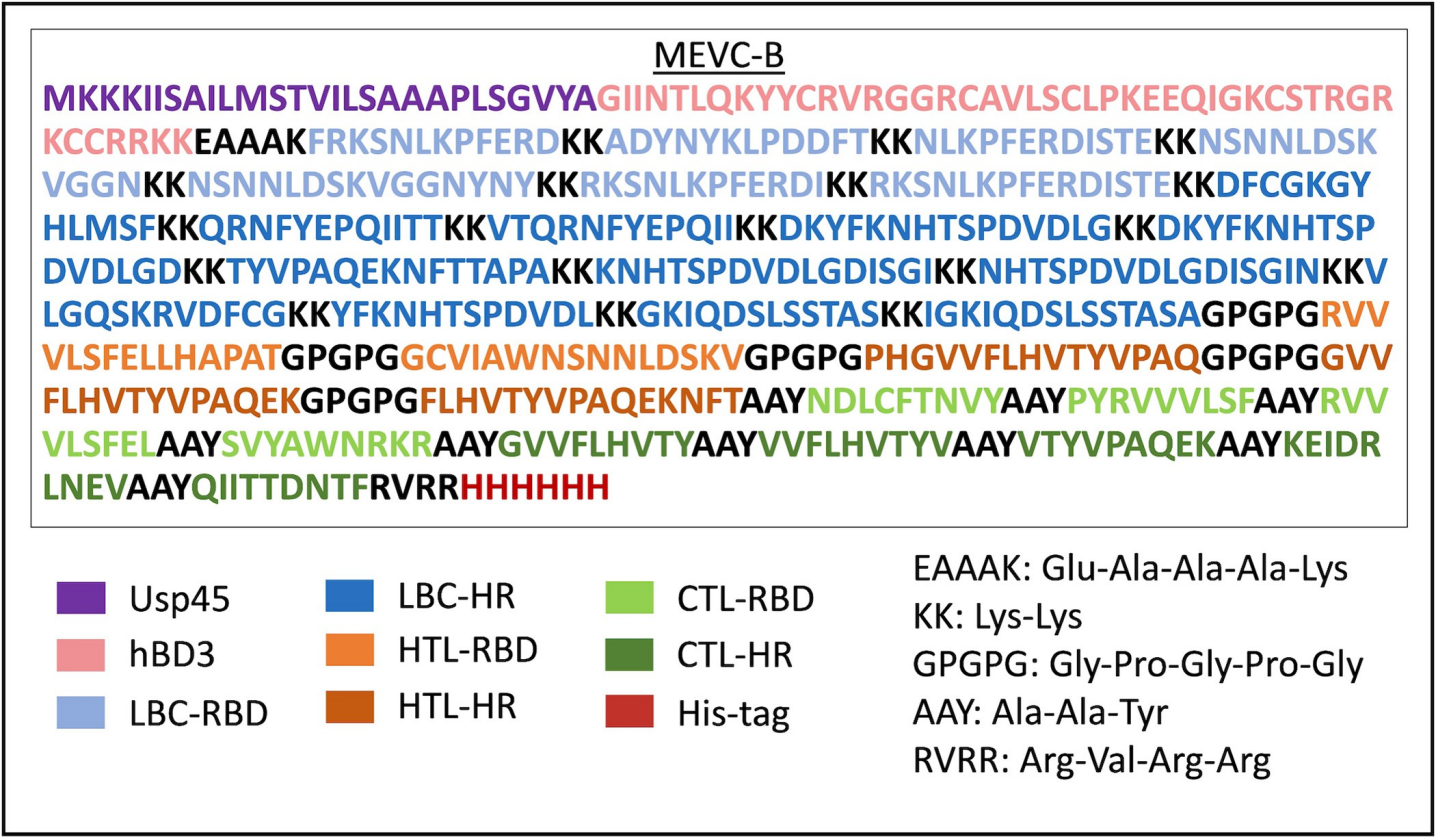
Multi-epitope vaccine construct B (MEVC-B) arrangement. The signal peptide, Usp45, was added before the hBD3 adjuvant. The EAAAK linker was used to connect the adjuvant and the LBC epitopes which were arranged first in the construct, epitopes from the receptor-binding domain (RBD) region followed by heptad repeat (HRs) region. The LBC epitopes were separated by KK linker. The HTL epitopes were joined with the GPGPG linker while the CTL epitopes were joined with the AAY linker. The epitopes were arranged from RBD region followed by epitopes from HRs region. The 6×His-tag was added to the C-terminal linked with RVRR linker.

Since only the arrangement of the epitopes of the MEVCs is different, the physicochemical properties, including the solubility score were similar for both MEVC-B (Fig 3) and MEVC-C. The MEVCs contained 558 amino acids with a molecular weight of 62.68 kDa, excluding the signal peptide as the signal peptide will be cleaved upon being released. A good antigenic protein should at least have a molecular weight of 40 kDa and up to 110 kDa in order to be immunogenic and targeted [82, 83]. The molecular weight of the MEVCs lies within the desired range for a target protein, making it an effective and highly antigenic target protein. The theoretical pI of the MEVCs is 9.73, indicating that our MEVC is basic. The instability index of the MEVCs is 26.79, which is considered stable (less than 40) [21], whereas the estimated value of the aliphatic index for the MEVCs is 68.80, indicating that the protein is thermostable [43]. The MEVCs also have a negative value of the GRAVY (-0.665) representing the hydrophilic nature of the subunit vaccine [43]. The estimated half-life of the MEVCs is calculated to be 30 hours in mammalian reticulocytes (*in vitro*), and *in vivo*, more than 20 hours in yeast, and more than 10 hours in *E*. *coli*. The physicochemical properties for the MEVC without the adjuvant was also checked for comparison. The MEVC without the adjuvant have similar properties except for its estimated half-life where the MEVC can only last for 1.1 hours in mammalian reticulocytes, 3 minutes in yeast and 2 min in *E*. *coli*. This indicated the present of the adjuvant is significant to ensure that the vaccine is stable and durable.

Overall, both our proposed arrangements of the MEVCs were considered to be stable in nature and have a long estimated half-life (more than 10 hours) [84]. The solubility of the MEVCs was calculated to be 0.475. A score of more than 0.450 indicates that the protein is highly soluble.

The physicochemical properties of the MEVCs predicted by the ProtParam server were compared with the experimental proven epitopes used for the construction of a novel antigenic multi-epitope vaccine against SARS-CoV-2 which has been immunologically tested from previous literature [85]. The physicochemical properties for the designed novel multi-epitope vaccine by Khairkhah *et al*. [85] was also predicted using ProtPram server. The antigenic multi-epitope vaccine had an average molecular weight and pI value of 25.26 kDa and 9.67, respectively. The instability and GRAVY indexes of the multi-epitope vaccine predicted with ProtParam server were 35.39 and -0.644, respectively [86], indicating that their vaccine construct is stable.

In addition, they also reported that the immunogenicity of the epitopes selected were also predicted using IgPred and IFNepitope web servers, and the epitopes that were predicted to had the ability to induce antibodies were included for the formation of their multi-epitope vaccine constructs [87]. Their vaccine constructs were predicted to have high solubility and stability to stimulate immunogenic reactions [87], similar to our designed MEVCs.

Further immunological study on the novel antigenic multi-epitope vaccine construct designed showed a significant stimulation of IgG, IgG2a, IFN-γ, TNF-α, IL-15, IL-21 and IL-6, and Granzyme B secretion in their animal studies with mice [85]. The similar characteristics predicted for the vaccine constructs by the ProtParam server supported the potential immunogenic responses that might be elicited by our MEVCs in further *in vitro* and animal studies.

#### Secondary and 3D structure of the MEVCs

Of the 558 amino acids in the MEVC-B arrangement, 111 (19.89%) were alpha helices, 140 (25.09%) formed extended strands, and 307 (55.02% of the MEVC structure) were composed of random coils. In the MEVC-C arrangement, 118 amino acids (21.15%) were alpha helices, 133 of 558 amino acids (23.84%) were extended strands, and the remaining 307 (55.02%) formed random coils. The secondary structure that dominated both MEVC-B and MEVC-C was the formation of random coils, which is unfavourable for the folding of the protein.

#### Validation and refinement of model structures

**Fig 4:**
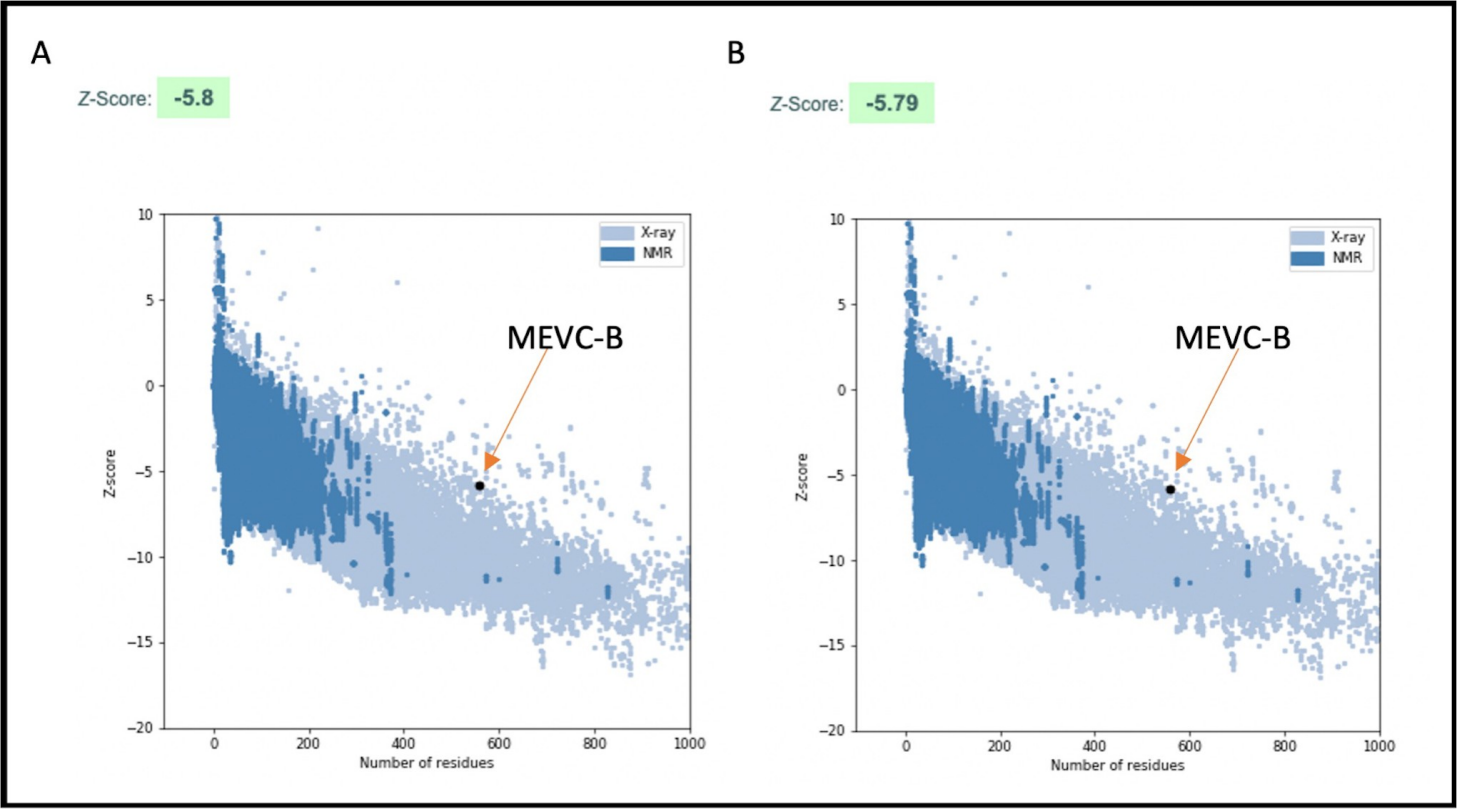
Pro-SA web server Z-score plot of 3D structure models for the MEVC-B. Before (panel A) and after (panel B) refinement with GalaxyRefine web server showed no significant difference in the Z-scores. The black dot in the blue shaded region represents our MEVC-B. The negative value of the Z-score indicates that the predicted 3D structure for the MEVC-B was reliable and stable as the predicted structure score value lies within the Z-scores of experimentally determined structures. Reprinted from ProSA server (https://prosa.services.came.sbg.ac.at/prosa.php) [46] under a CC BY license, with permission from PLOS ONE, original copyright 2023.

**Fig 5:**
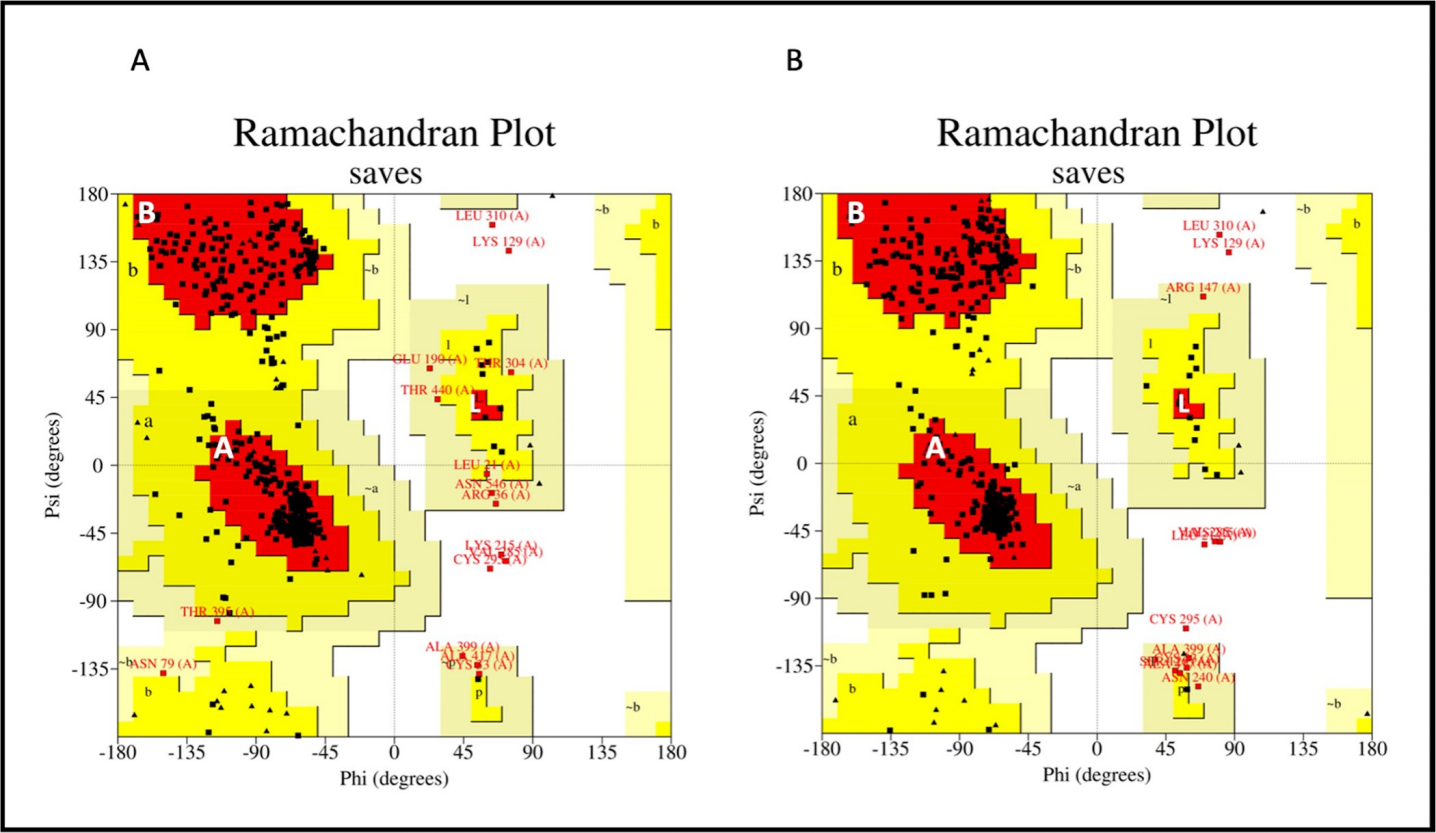
Ramachandran plot of 3D structure models for MEVC-B. Plot from panel A showed the Ramachandran plot before refinement and panel B for the refined model structures generated with PROCHECK web server. The most favoured regions were labelled in white letters “A”, “B”, “L”. The black squares and triangles represent the residues that were within favourable regions. Red squares represent residues that were within the disallowed region of the Ramachandran plot. The residues were slightly more concentrated in the most favoured region and also had slightly more residues within the disallowed region after refinement compared to before refinement. A good quality model structure is expected to have more than 90% residues [47]. Reprinted from PROCHECK web server (https://www.ebi.ac.uk/thornton-srv/software/PROCHECK/) [47] under a CC BY license, with permission from PLOS ONE, original copyright 2023.

Therefore, each of the five model structure of MEVC-B and MEVC-C was subjected to validation with the ProSA and PROCHECK web servers, and refinement with GalaxyRefine tool. Model 2 of the MEVC-B arrangement has both a negative Z-score (-5.80) and lies within the blue dots plotted area (Fig 4A). The Ramachandran plot showed that 86.8% of residues of model 2 were in the most favourable region (Fig 5A and S5 Table) compared to other four models. The quality factor of more than 95 from ERRAT was determined based on the characteristic of atomic interaction verifying that the structure characteristic of atomic interaction [88]. However, none of our model structures passed the good quality score for the ERRAT and Ramachandran plots (S5 Table).

Model 2 of the MEVC-B with the highest residues within the most favourable region and least residues percentage in the disallowed region was further refined. The refinement generated another five refined models with improved Rama-favoured regions compared to the initially submitted model 2 (S5 Table). Refined model 4 have good Rama-favoured region (94.1), GDT-HA (0.9745), RMSD (0.370) and MolProbity (1.903) (S6 Table). Thus, the refined model 4 of MEVC-B was input into ProSA and PROCHECK servers for re-validation [77]

The Z-score of the refined model 4 of MEVC-B was a negative value (-5.79) (Table 4) and was within the range of Z-scores found for native proteins with similar sizes (Fig 4B), suggesting that the refined model structures predicted for MEVC-B was reliable [46, 89]. The Ramachandran plot generated from the PROCHECK server for the refined model structure showed that 91.1% of the residues were within the most favourable region, 6.1% were in the additional allowed regions, 1.2% were in the generously allowed regions, and the remaining 1.2% of the residues were exhibited in the disallowed regions (Fig 5B). The results indicated that the refined model structure of MEVC-B was the best model structure for the vaccine construct and was used for molecular dockings (Table 4).

**Table 4:** Re-validation of five models structures of model 2 MEVC-B generated by GalaxyRefine with ProSA, ERRAT and PROCHECK web servers.

Model	ProSA		ERRAT	Ramachandran plot	
	Z-score	Region		Most favoured region	Disallowed region
Model_1	-5.72	Within blue region	82.7	90.7	1.2
Model_2	-5.79	Within blue region	86.7	90.3	1.6
Model_3	-5.82	Within blue region	86.5	90.1	1.4
Model_4	-5.79	Within blue region	86.7	91.1	1.2
Model_5	-5.87	Within blue region	84.6	89.7	1.2

#### Molecular docking and binding interactions with TLR molecules

The ClusPro docking server generated 30 different clusters of members along with their interaction energies. The cluster with the highest number of members was ranked as the top cluster. The top five docked MEVCB-TLR4-MD-2 complex clusters had the lowest energy scores of -1228.4, -1203.8, -1173.1, -1207.1 and -1122.3, ranked from the first to the fifth cluster, respectively. The docking between the MEVC-B and TLR1-TLR2 complex generated the top five clusters with the lowest energy score of -1115.7, -1197.3, -1145.8, -1179.4 and -1032.0. The top cluster with the highest number of members and the most negative lowest energy score was analysed for its binding interactions [22, 23].

The binding affinity of the docked MEVCB-TLR4-MD-2 complex ranked first has the lowest free binding energy, -23.9 kcal mol^-1^, while for MEVCB-TLR1-TLR2, the free binding energy of the top ranked docked complex was -18.9 kcal mol^-1^. A more negative free binding energy, -28.5 kcal mol^-1^, was calculated for the fifth cluster of the MEVCB-TLR1-TLR2 complex, hereafter known as MEVCB-TLR1-TLR2-b, was also selected for further analysis. The negative binding affinity score showed that the binding interaction between the MEVC and the TLR molecules was favourable. This suggested that the MEVC design could effectively bind with the TLR molecules, which are involved in triggering appropriate immune responses against SARS-CoV-2.

**Fig 6:**
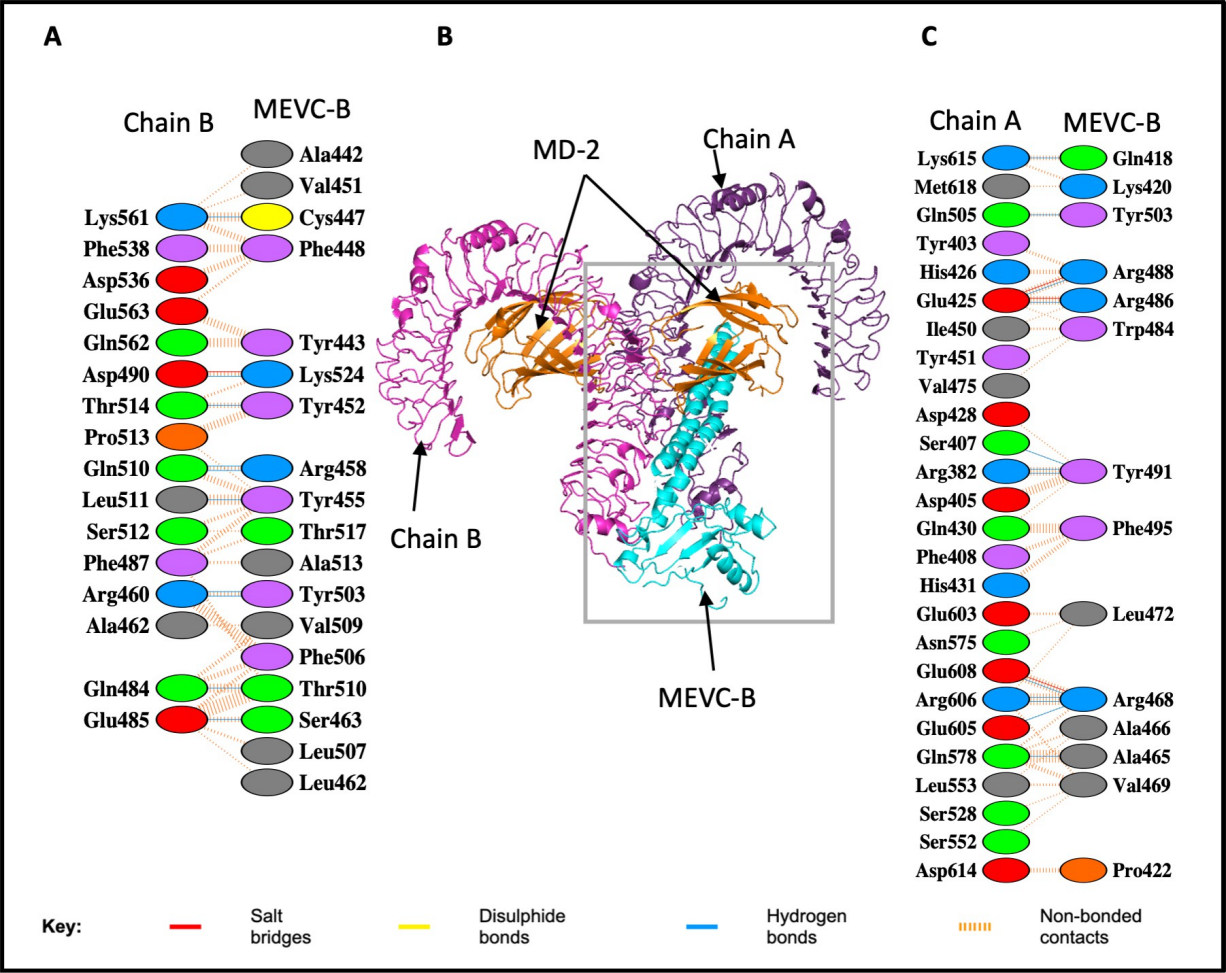
Molecular docked model structure of the MEVCB-TLR4-MD-2 complex. Panel B showed the homodimer TLR4, labelled as Chain A (purple) and Chain B (magenta), the MD-2 structures coloured in orange and MEVC-B structure in cyan. Panel A and C showed the binding interactions between the docked complex. The MEVC-B structure interacted with both chains of TLR4 (panel A and C) but only one chain of the MD-2 in the docked complex (not shown). Reprinted from PDBsum Generate web server (https://www.ebi.ac.uk/thornton-srv/databases/pdbsum/Generate.html) [72] under a CC BY license, with permission from PLOS ONE, original copyright 2023.

The binding contacts between the MEVCB-TLR complexes were analysed using PDBsum and PRODIGY web servers to assess if the binding interactions were occurring within the TLR molecule binding domains for effective immune response to be triggered. The LRRs motif within the TLR4 molecule plays an important role in the recognition of PAMP. In the protein-protein interaction analysis with PDBsum server, 26 residues from chain A of the TLR4 molecule were interacting with 14 residues from the MEVC-B (Fig 6C), and from the chain B of the TLR4 molecule, 16 residues were interacting with 18 residues of the MEVC-B (Fig 6A). Most of the residues from TLR4 molecules that were involved in the binding with the MEVC-B were within the LRRs motif, and the MEVC-B was also interacting with one chain of the MD-2 molecule (not shown). This indicated that the MEVCB-TLR4-MD-2 complex could promote downstream signalling and stimulate immune responses.

The binding affinity between the complexes which was calculated by PRODIGY web server was based on the intermolecular contacts at the interface of the complexes within the threshold distance of 5.5 Å [54, 76, 77]. Therefore, we compared the intermolecular contacts from the PRODIGY and the interacting residues generated form PDBsum web server. In the MEVCB-TLR4-MD-2 complex, there were 42 residues from chain A of TLR4 molecule interacting with 28 residues from the MEVC-B where 26 of the 42 residues from chain A of TLR4 and 14 of 28 residues from MEVC-B were consistent with the interacting residues identified from PDBsum (S7 Table). Meanwhile, the PRODIGY server identified 29 residues in chain B of TLR4 molecule interacted with 24 residues of the MEVC-B where all the interacting residues identified from PDBsum were also consistent (S7 Table). The PRODIGY server also identified that only one chain of the MD-2 molecule was interacting with the MEVC-B as in the PDBsum (not shown).

**Fig 7:**
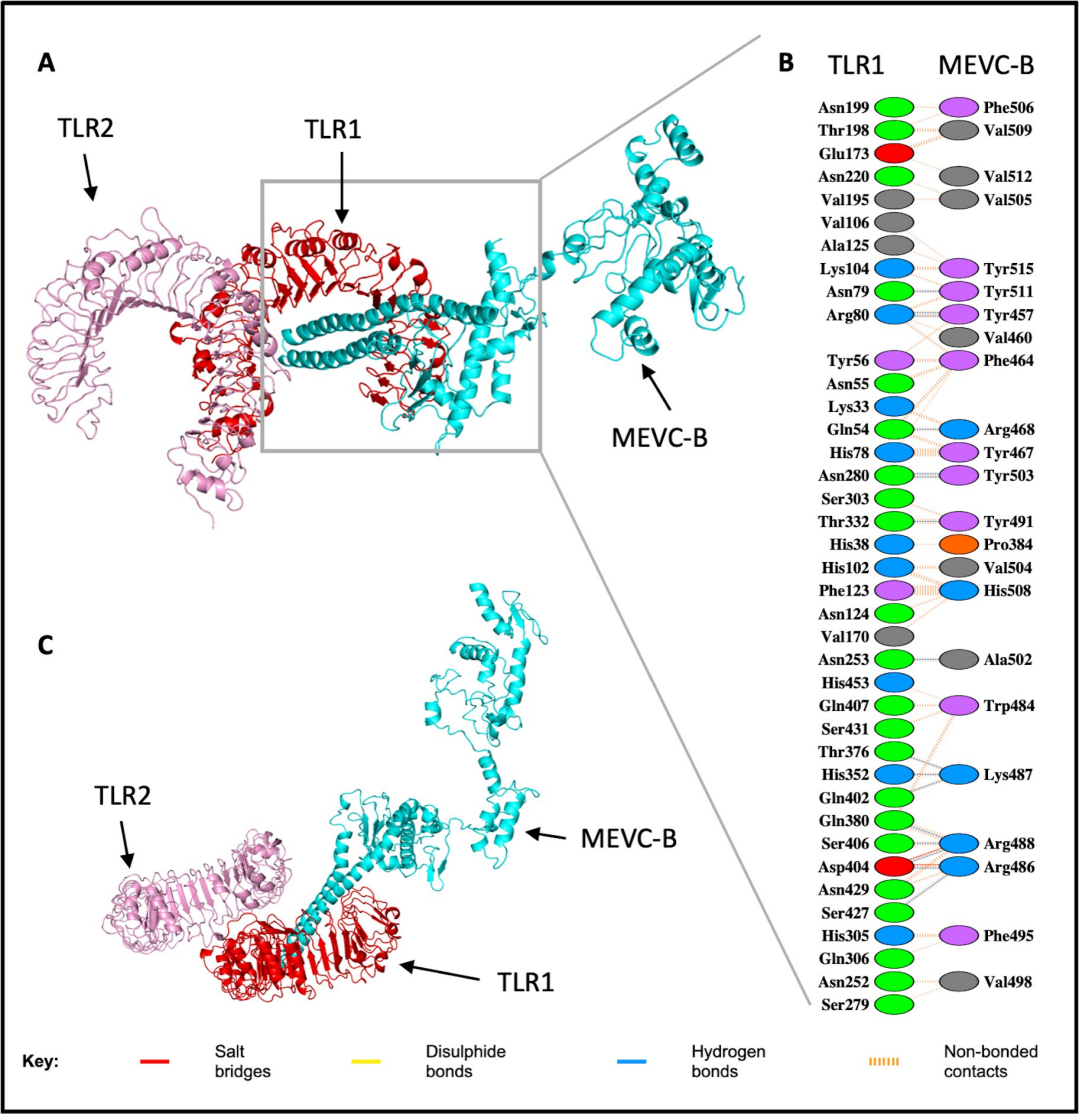
Molecular docked model structure of the MEVCB-TLR1-TLR2 complex. The docked complex showed that there is no interaction predicted between the MEVC-B and TLR2 chain in protein-protein complex (panel A). The binding interactions between TLR1 of the TLR1-TLR2 heterodimer (red) and MEVC-B (cyan) were visualised with Pymol and PDBsum server in Panel B. The interaction within the complex is only limited between the MEVC-B with TLR-1 which is shown in the top-view of the docked complex (panel C). Reprinted from PDBsum Generate web server (https://www.ebi.ac.uk/thornton-srv/databases/pdbsum/Generate.html) [72] under a CC BY license, with permission from PLOS ONE, original copyright 2023.

**Fig 8:**
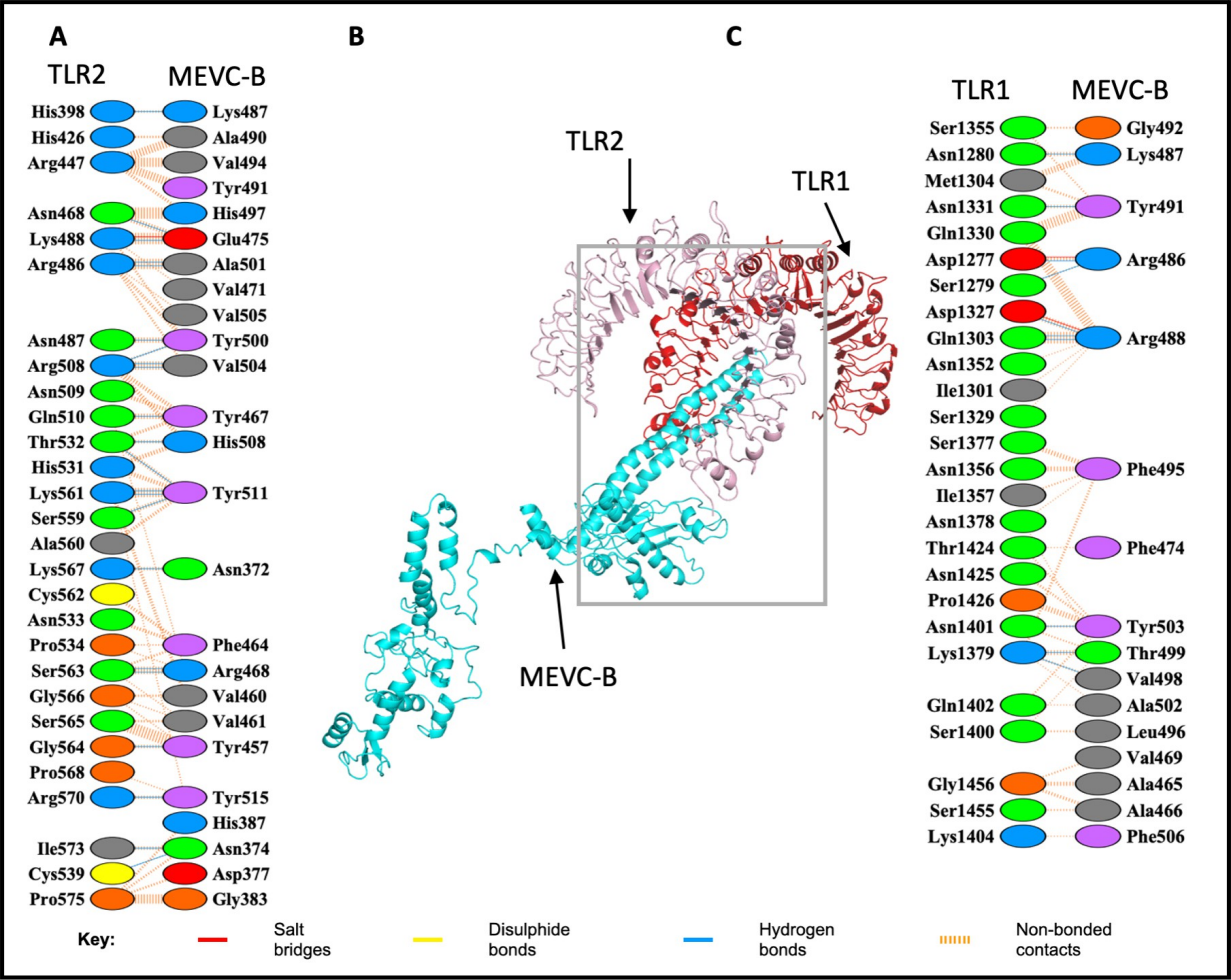
Molecular docked model structure of the MEVCB-TLR1-TLR2b complex. The docked complex showed that the interaction between the MEVC-B with TLR1 (panel C) and TLR2 chain (panel A). The binding interactions between the TLR1-TLR2 heterodimer (red and pink) and MEVC-B (cyan) were visualised with Pymol and PDBsum server in Panel B. The binding residues in the interaction were not included in the reported ligand-binding residues (panel B and C). Reprinted from PDBsum Generate web server (https://www.ebi.ac.uk/thornton-srv/databases/pdbsum/Generate.html) [72] under a CC BY license, with permission from PLOS ONE, original copyright 2023.

The binding interaction analysis of the MEVCB-TLR1-TLR2 complex showed that the interacting residues of the MEVC-B molecule were only limited to the TLR1 molecule (Fig 7A and 7C). The interacting residues visualisation generated by the PDBsum server showed that 39 residues from TLR1 molecule were involved in the interaction with 23 residues of MEVC-B, but only 6 of them were the reported ligand-binding residues (Asn280, Ser303, Thr332, His305, Gln306 and Ser279) [75] (Fig 7B and S8 Table). For the MEVCB-TLR1-TLR2-b complex with the most negative binding energy score, 26 residues from TLR1 were interacting with 16 MEVC-B residues while 28 residues from TLR2 interacted with 25 residues from MEVC-B molecule (Fig 8). However, none of the identified interacting residues were the reported ligand-binding residues.

In comparison with the intermolecular contacts at the interface of the MEVCB-TLR1-TLR2 complex, 62 residues from TLR1 molecule that were interacting with 33 residues from MEVC-B identified by PRODIGY were consistent with the 39 residues from TLR1 molecule and the 23 residues from MEVC-B identified by PDBsum web server (S8 Table). The MEVCB-TLR1-TLR2-b showed that both TLR1 and TLR2 molecules were interacting with the MEVC-B. A total of 34 interacting residues from TLR1 molecule were interacting with 25 residues from MEVC-B, while 41 residues of TLR2 interacted with 43 residues from MEVC-B. For the MEVCB-TLR1-TLR2-b complex, all 26 and 28 interacting residues of TLR1 and TLR2, respectively, identified from PDBsum were consistent with residues from PRODIGY server. Similarly, for the interacting residues listed for MEVC-B molecule.

The binding interaction analysis from the PRODIGY web server indicated that the intermolecular interacting residues that were in contact within the complexes were coherent to the results output from the PDBsum server. The highly negative free binding energy calculated for the complexes generated from both docking servers indicated that the binding interaction between the MEVC-B with TLR4 homodimer and TLR1-TLR2 heterodimer molecules was highly likely to occur.

To validate the docking results from ClusPro, further docking was performed using HADDOCK web server. We used the surface interacting residues identified using PRODIGY web servers between the protein-protein complex as input to run the docking with HADDOCK. The HADDOCK server generated 10 different clusters of members along with their interaction energies. In this server, the cluster with the most negative HADDOCK was ranked the most top regardless of the number of members in the clusters.

The docked complex of MEVCB-TLR4-MD-2 generated from HADDOCK web server had a HADDOCK score of -96.2 +/- 10.4, -92.7 +/- 31.6, -92.5 +/- 8.6, -86.0 +/- 12.8 and -85.6 +/- 11.4, ranked from the first to the top five clusters, respectively (Table 6). The HADDOCK scores of the top five clusters generated from HADDOCK for MEVCB-TLR1-TLR2 were -131.2 +/- 16.7, -129.5 +/- 15.3, -122.8 +/- 9.2, -119.5 +/- 14.0 and -89.1 +/- 8.8, respectively (Table 6). The docked complexes with of the top five ranked clusters generated from HADDOCK were calculated for their free binding energies and compared with docked complex from ClusPro (Tables 5 and 6). From the free binding energy analyses using PRODIGY web server, the docked complexes generated from the blind docking using ClusPro web server had the lowest free binding energy indicating the highest interaction and strongest affinity between the vaccine constructs and both the homodimer TLR4 and heterodimer TLR1-TLR2 molecules. Thus, the docked complexes generated from the blind docking from ClusPro web server were used for further structural stability and flexibility analyses through molecular dynamic simulations.

**Table 5:** Docking results of the top five cluster with the most negative lowest energy scores the on the docked complexes between MEVC-B and TLR molecules generated by ClusPro web server and the binding affinity between the complex.

Complex	Cluster	Cluster Size	Lowest Energy Score	Free binding energy (kcal mol^-1^)
MEVCB-TLR4-MD-2	0	37	-1228.4	-23.9
	1	34	-1203.8	-19.5
	2	34	-1173.1	-19.5
	3	33	-1207.1	-18.7
	4	32	-1122.3	-16.5
MEVCB-TLR1-TLR2	0	63	-1115.7	-18.9
	1	50	-1197.3	-20.9
	2	47	-1145.8	-18.2
	3	32	-1179.4	-16.0
	4	30	-1032.0	-28.5

**Table 6:** Docking results of the docked complexes between MEVC-B and TLR molecules for the top five cluster with the Z-score and HADDOCK score generated by HADDOCK web server and the binding affinity between the complex.

Complex	Cluster	Cluster Size	HADDOCK Score	Z-Score	Free binding energy (kcal mol^-1^)
MEVCB-TLR4-MD-2	5	9	-119.8 +/- 11.8	-2.0	-15.7
	7	8	-108.0 +/- 10.8	-1.4	-14.9
	1	26	-96.5 +/- 0.7	-0.8	-13.4
	2	16	-79.5 +/- 6.4	0.1	-13.5
	4	11	-76.5 +/- 5.8	0.2	-13.7
MEVCB-TLR1-TLR2	1	24	-131.2 +/- 16.7	-1.4	-12.2
	19	4	-129.5 +/- 15.3	-1.4	-13.0
	3	13	-122.8 +/- 9.2	-1.1	-12.7
	8	9	-119.5 +/- 14.0	-0.9	-11.1
	12	6	-89.1 +/- 8.8	0.5	-10.3

For each of the individual epitope docked with the TLR molecules using ClusPro web server, epitope ^1062^FLHVTYVPAQEKNFT^1076^, one of the HTL epitopes included in the MEVC-B has the most negative free binding energy (-19.9 kcal mol^-1^) with TLR4 homodimer molecule (Table 7). From the docking with HADDOCK, the epitope also has a strong binding affinity with the TLR4 molecule where the free binding energy is -13.0 kcal mol^-1^ (S9 Table). Nevertheless, from the docked protein-protein complex, MEVCB-TLR4-MD-2, the ^1062^FLHVTYVPAQEKNFT^1076^ epitope was not seen to be interacting with any of the molecules from the homodimer TLR4-MD2 but the Gln1071 (Gln418 in Fig 6) and Lys1073 (Lys420 in Fig 6) residues were identified as two of the interacting residues with the TLR4 chain (Fig 6).

**Table 7:** Docking results of the individual epitopes and TLR molecules for the top 10 highest binding affinity and the most negative lowest energy scores generated by ClusPro web server.

Complex	Epitope Number *	Epitope	Lowest Energy Score	Free binding energy (kcal mol^-1^)
MEVCB-TLR4-MD-2	24	^1062^FLHVTYVPAQEKNFT^1076^	-1187.0	-19.9
	23	^1059^GVVFLHVTYVPAQEK^1073^	-1300.6	-19.2
	15	^1158^NHTSPDVDLGDISGIN^1173^	-989.8	-18.2
	7	^457^RKSNLKPFERDISTE^471^	-904.8	-17.5
	4	^37^NSNNLDSKVGGN^448^	-1284.3	-16.8
	19	^931^IGKIQDSLSSTASA^944^	-1036.6	-16.8
	21	^431^GCVIAWNSNNLDSKV^445^	-1360.2	-16.5
	13	^1066^TYVPAQEKNFTTAPA^1080^	-1189.5	-16.1
	33	^1113^QIITTDNTF^1121^	-1034.3	-16.1
	9	^1106^QRNFYEPQIITT^1117^	-1160.7	-15.8
MEVCB-TLR1-TLR2	4	^437^NSNNLDSKVGGN^448^	-539.8	-20.5
	33	^1113^QIITTDNTF^1121^	-802.1	-19.7
	21	^431^GCVIAWNSNNLDSKV^445^	-996.1	-19.2
	18	^932^GKIQDSLSSTAS^943^	-508.9	-18.8
	20	^509^RVVVLSFELLHAPAT^523^	-615.3	-17.0
	26	^507^PYRVVVLSF^515^	-1056.6	-16.2
	2	^419^ADYNYKLPDDFT^430^	-797.9	-16.0
	23	^1059^GVVFLHVTYVPAQEK^1073^	-1053.1	-15.7
	22	^1057^PHGVVFLHVTYVPAQ^1071^	-933.9	-15.3
	28	^349^SVYAWNRKR^357^	-818.3	-15.3

*Based on arrangement in the MEVC-B in Fig 3.

As for the binding with TLR1-TLR2 molecule, another HTL epitope from the 33 epitopes included in the MEVC-B, ^507^PYRVVVLSF^515^, has a low binding free energy (-16.2 kcal mol^-1^) with the TLR1 molecule from the TLR1-TLR2 heterodimer indicating a strong and favourable binding the two molecules. From the MEVCB-TLR1-TLR2 complex, the Tyr508, Val511, and Phe514 from the ^507^PYRVVVLSF^515^ HTL epitope, are the identified interacting residues involved in the binding with TLR1 (Tyr457, Val460 and Phe464 in Fig 7, respectively). The Gln1071 and Tyr508 residues have a potential to be identifies as the critical binding epitopes in the protein-protein complex as they formed hydrogen bonding with the residues from the TLR4 and TLR1 molecule, respectively.

#### Structural stability and flexibility of the docked protein-protein complexes

**Fig 9:**
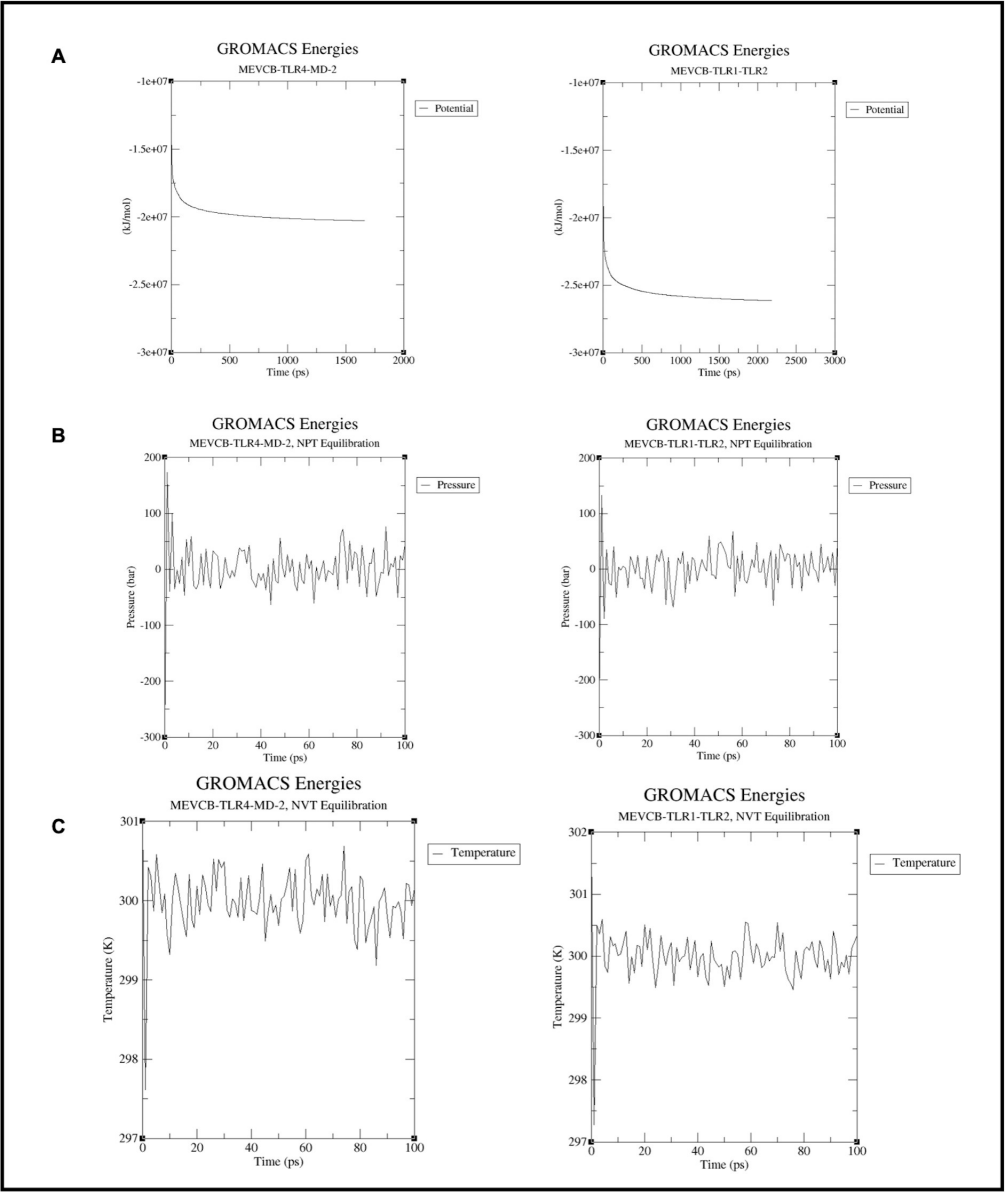
Energy minimisation and equilibration of the system for MD simulations. The potential energy of the system of both docked complexes were minimised prior to the molecular dynamic simulations and the plots showed that the minimum potential energy of both docked complexes were stable over the course of 100-ps simulation (panel A). The system in both protein-protein complex was equilibrated under two phases: pressure (NPT) and temperature (NVT) ensembles. The graph of the NPT and NVT equilibration phases for the protein complexes in panel B and C, respectively, showed that the system in both protein complexes were instantly stabilised and reached the desired pressure and temperature. The graphs were plotted using the XMGRACE plotting tool [71].

The structural stability and flexibility of the MEVC-B and the TLR molecules were estimated and assessed through the molecular dynamic (MD) simulations using the GROMACS software. Before the dynamic simulations were run on both the protein-protein complexes, MEVCB-TLR4-MD-2 and MEVCB-TLR1-TLR2, an energy minimisation was conducted to ensure that the simulation system has no steric clashes or inappropriate geometry by evaluating the potential energy (kJ mol^-1^) and the maximum force of the system [70]. A potential energy less than 1000 kJ mol^-1^ indicates that the system is stable and ready for simulation. From the energy minimisation of 50,000 steps, the potential energy of the system for MEVCB-TLR4-MD-2 complex was calculated to be -2.0 e + 07 kJ mol^-1^, while for MEVCB-TLR1-TLR2, the minimum potential energy was -2.5 e + 07 kJ mol^-1^, indicating that the system was stable (Fig 9A). After the equilibration step, both protein-protein complexes showed that the pressure values were fluctuated from the reference value (1 bar) over the course of 100-ps equilibration phase while the temperature of the system reached the target value of 300K in the 100-ps equilibration phase (Fig 9B and 9C).

**Fig 10:**
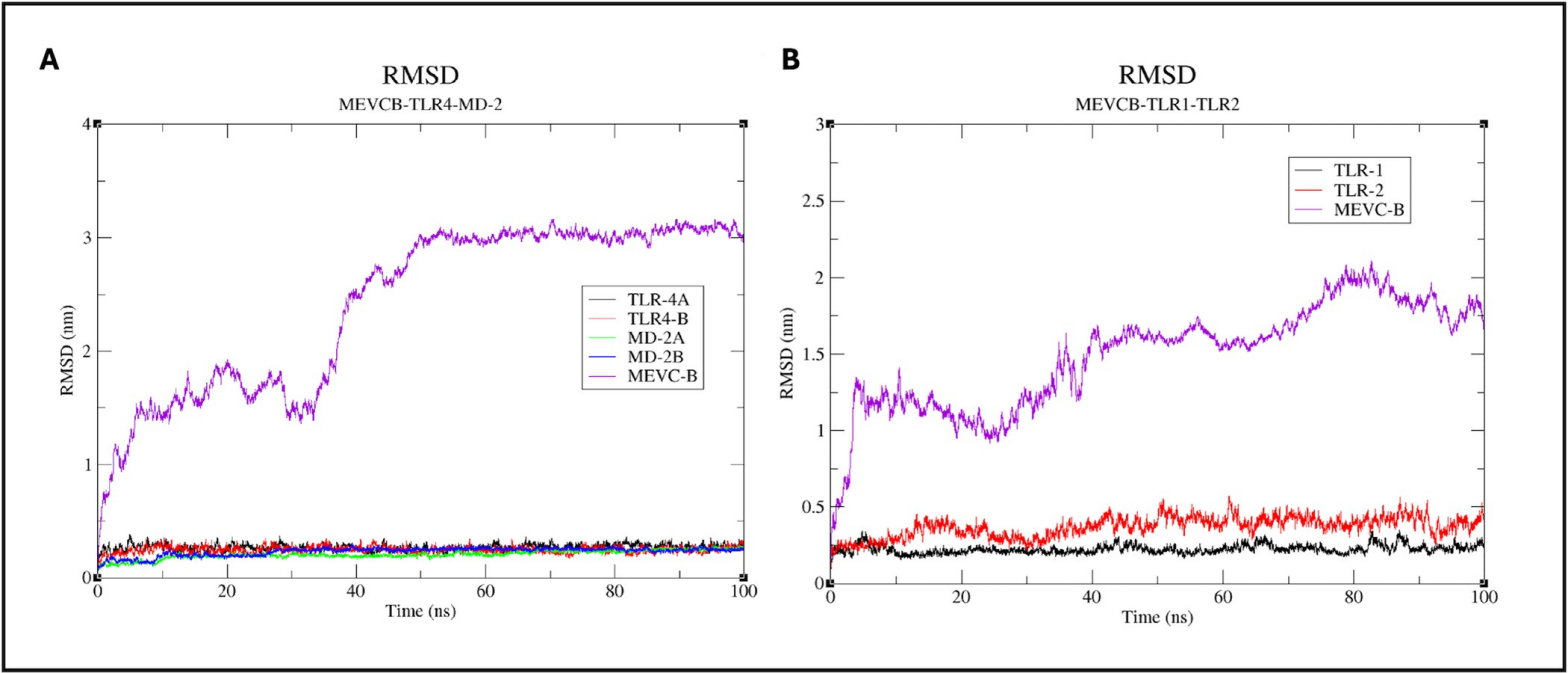
RMSD calculations of the docked protein complexes from MD simulations. The RMSD calculation of C-alpha atoms of each chain in the MEVCB-TLR4-MD-2 (Panel A) and MEVCB-TLR1-TLR2 (Panel B) complex. The RMSD value assessed the structural stability of the protein in the complexes over the 100ns MD simulations. The graphs were plotted using the XMGRACE plotting tool [71].

To assess the structural stability and flexibility of the complexes, trajectory analyses were performed after the 100ns simulations, and the RMSD and RMSF values of the carbon atoms of each chain in the complex were plotted in Fig 10. The plot for the RMSD values of the MEVCB-TLR4-MD-2 complex showed that the carbon atoms of the proteins in the complex were stabilised after 50ns at 3nm with mild fluctuations (Fig 10A). However, from the MD simulation on the MEVCB-TLR1-TLR2 complex, the RMSD values had high-peak fluctuations indicating that the proteins were not stabilised over the course of 100-ns. A longer simulation time might be needed to allow the protein-protein complex to reach a more stable phase (Fig 10B). Nevertheless, from the five different timepoints within the MD simulation of the complex, the MEVCB and TLR1-TLR2 molecule still remained in contact with each other and similarly to the MEVCB-TLR4-MD-2 complex, the number of residues from the MEVCB interacting with TLR1-TLR2 molecule increases with time.

**Fig 11:**
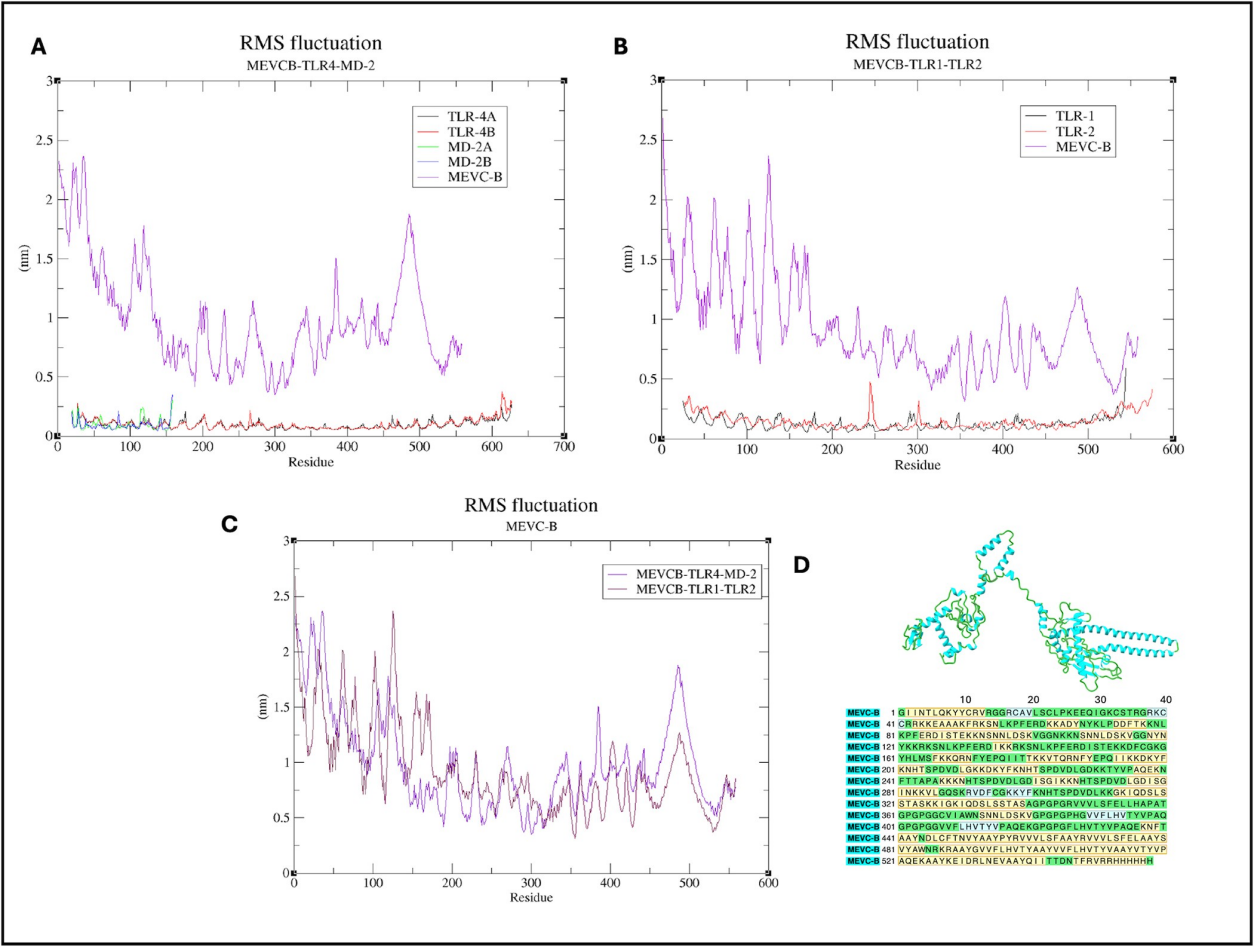
RMSF calculations of the C-alpha atom of each chain in the docked protein complexes from MD simulations. The RMSF of proteins in the MEVCB-TLR4-MD-2 (Panel A) and MEVCB-TLR1-TLR2 (Panel B) to show the flexibility of the C-alpha atom of each residues in all the chains of the protein-protein complexes. The RMSF of the MEVC-B from both complexes were compared (Panel C) to identify the high peak regions in the plot which were caused by the loop regions in the MEVC-B structure (Panel D). The graphs were plotted using the XMGRACE plotting tool [71].

The RMSF graph for each protein in both complexes showed that the atoms in the MEVC-B are highly flexible with RMSF values between 0.3 to 3.0nm as compared to the molecules of the TLR4-MD-2 homodimer and TLR1-TLR2 heterodimer with lower RMSF value ranging from 0 to 0.5nm (Fig 11A and 11B). Since there are many loop regions in the MEVC-B structure (Fig 11D), many high peaks were seen in the RMSF graph of the MEVC-B in both complexes (Fig 11C).

The free binding energies of the MEVCB-TLR4-MD-2 and MEVCB-TLR1-TLR2 at five timepoints - 20ns, 40ns, 60ns, 80ns and 100ns—were also assessed using PRODIGY web server and compared to each other along with the free binding energy of the original docked complex (0ns) in Table 8. The free binding energy calculated between the MEVC-B and the TLR molecules was only slightly different for each timepoint throughout 100ns MD simulations where at 100ns, the last timepoint of the simulation, the binding free energy was the highest for both complexes. Although for MEVCB-TLR4-MD-2 complex the 0ns (original docked complex) has higher free binding energy as compared to at 100ns, the number of interacting residues from the MEVC-B increases. For MEVCB-TLR1-TLR2 complex, free binding energy of the complex increased as the timepoint increased except for timepoint at 40 and 60ns. The number of residues from MEVC-B interacting with the TLR1-TLR2 heterodimer molecule in the complex increased as the timepoint increased and the highest number of interacting residues is at 100ns. The analysis from the trajectory files also did not show any proteins unfolding at any timepoint of the simulation (not shown) and it is expected that the interaction between the vaccine construct and the TLR molecules is favourable and is likely to occur.

**Table 8:** Analysis on the binding interactions at different time frame during the 100ns MD simulation of MEVCB-TLR4-MD-2 and MEVCB-TLR1-TLR2.

Complex	Timepoint (ns)	Free binding energy (kcal mol^-1^)	Number of MEVC-B residues interacting with the chains of the TLR molecules
MEVCB-TLR4-MD-2	0	-23.9	52
	20	-18.1	51
	40	-18.7	59
	60	-19.6	58
	80	-20.1	63
	100	-20.5	65
MEVCB-TLR1-TLR2	0	-18.9	33
	20	-19.5	38
	40	-18.5	41
	60	-18.3	41
	80	-18.9	43
	100	-20.0	47

**Fig 12:**
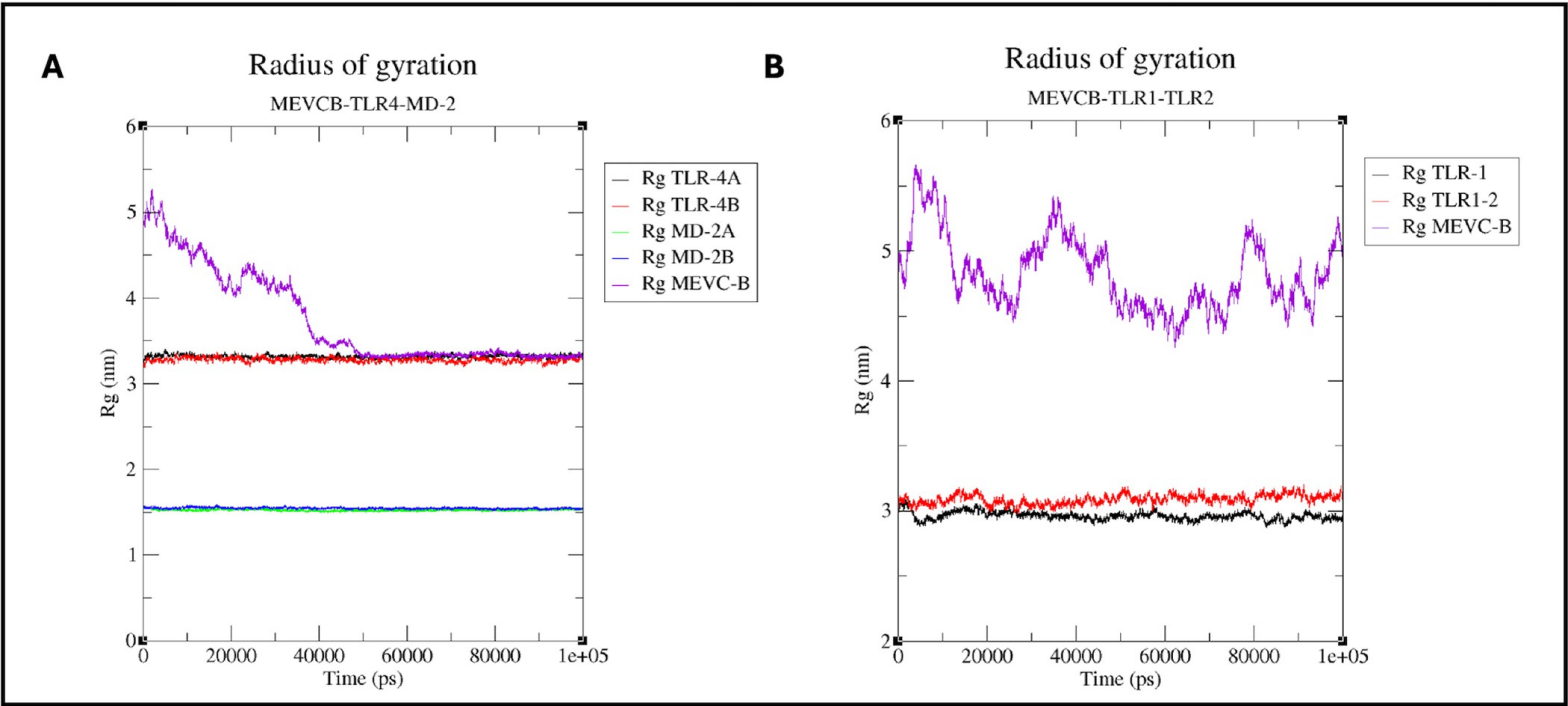
Radius of gyration (Rg) values of the proteins. The compactness of the proteins in MEVCB-TLR4-MD-2 (Panel A) and MEVCB-TLR1-TLR2 (Panel B) complexes were calculated using GROMACS and visualised in Rg value. The graphs were plotted using the XMGRACE plotting tool [71].

To measure the compactness of the proteins in the complex, the radius of gyration (Rg) value for the protein-protein complex from the simulation was plotted in Fig 12. The plot for the MEVCB in MEVCB-TLR4-MD-2 complex showed that the designed protein did undergo large conformational changes at the start of the simulation (0-50ns) as can be seen from the high fluctuations of the graph. After 50ns simulation, the Rg value of the MEVC-B in the complex decreased to similar Rg value as the TLR4 chains at 3.3nm, with mild fluctuations indicating that the MEVC-B is tightly packed after 50ns of simulation and there might be only small conformational changes occurring in the proteins in the complexes (Fig 12A). However, the Rg plot of the MEVC-B in complex MEVCB-TR1-TLR2, showed high fluctuations indicating very large conformational changes compared to TLR1 and TLR2 (Fig 12B). Similarly, longer simulations may be needed for MEVCB-TLR1-TLR2 to allow the complex to reach a more stable and flexible phase as what was observed in MEVCB-TLR4-MD-2. Nevertheless, we can conclude that the MEVC-B may not fold easily and has high potential to be able to interact with the TLR4 molecule.

#### Codon optimisation

To maximise the expression of the MEVC-B *in vitro*, the MEVC was codon optimised for our chosen host, *L*. *lactis*. The MEVC-B sequence, which include the signal peptide, Usp45, and the 6×His-tag at the N- and C-terminal, respectively, was codon optimised for *L*. *lactis subsp*. *cremoris* MG1363. The DNA sequence of the MEVC was comprised of 1755 bp, and the codon adaptation index (CAI) calculated for the MEVC was 1.0 with 30.8% GC content. The results indicated that the MEVC can be efficiently expressed in *L*. *lactis* NZ3900, which is a derivative of the MG1363.

## Discussion

Although the spread of the SARS-CoV-2 virus within the human population is no longer a global threat, its variants are still circulating and have not been completely eradicated [9]. While numerous COVID-19 vaccines have been developed and approved, this study explores an alternative approach for the development of longer lasting mucosal vaccines against the SARS-CoV-2 virus and its variants.

The S protein of SARS-CoV-2, particularly the RBD region, which recognises the host receptor ACE2 for its entry mechanism into host cells [90–92], and the HR1 and HR2 regions [93–98], which mediate the fusion of viral entry, are highly conserved among other SARS CoV-2 variants [25, 99–103] and have been extensively targeted for vaccines development [1, 21, 82, 84, 104–111]. Multi-epitope COVID-19 vaccines targeting these highly conserved regions could contribute to stronger and longer-lasting immune reactions, as they stimulate concurrent humoral and cellular responses [82]. Therefore, a multi-epitope-based oral vaccine targeting the highly conserved regions of the S protein, particularly the RBD and HR1-HR2 domains, would be more effective to target the existing and emerging variants.

In this study, we explored the S protein of the SARS-CoV-2 virus, in particular the RBD and the HR1-HR2 domains of the S protein, to develop a mutational resistant multi-epitope oral vaccine against SARS-CoV-2 variants *in silico* using various immunoinformatic tools. There were different potential multi-epitope vaccine constructs that have been designed using immunoinformatic approaches against diseases and pathogens such as *Streptococcus agalactiae* [112] and dengue [113], which have shown promising results. The identification and prediction of the crucial epitopes for the formulation of peptide-based vaccines have been greatly improved, owing to the availability of current advanced bioinformatics tools such as the NetCTLpan v1.1, NetMHCIIpan v4.1 and IEDB Analysis Resource. Vaccine constructs formulated with highly immunogenic and antigenic peptides with a reduced risk of developing unwanted reactions are important criteria for the successful development of safe and effective vaccines.

Our study discovered that only a small portion of the epitopes within the RBD region that met all of our selection criteria and were highly conserved compared to the epitopes predicted within the HR1-HR2 region of the S protein. A total of 9 CTLs, 5 HTLs and 19 LBC epitopes were finalised and selected for the formation of the MEVCs. The MEVCs consisted of the 33 T and B lymphocyte epitopes were joined by associated linkers, a signal peptide, Usp45, at the N-terminal of the MEVCs, to allow secretion of the heterologous protein by our selected *Lactococcus* host, the *L*. *lactis*, and a 6×His-tag at its C-terminal for ease of protein purification (Fig 3).

In the evaluation study of the three different arrangements of the epitopes in the MEVCs (MEVC-A, MEVC-B, MEVC-C), the physicochemical properties and structure analyses showed that the MEVC-B was a better vaccine construct candidate compared to MEVC-A and MEVC-C. MEVC-B was predicted to be a probable antigen, non-toxic, non-allergenic and highly soluble. MEVC-B also had a high antigenicity value (0.7081) and was categorised as a probable antigen. The solubility index for MEVC-B (0.475) was also above the average threshold for soluble protein (0.450), indicating that the heterologous protein expressed from the vaccine construct was sufficient to promote efficient folding and prevent protein aggregation [114].

A 45-mer adjuvant, the human β-defensin 3 (hBD3), was also added to our MEVCs as it was reported that β-defensins can enhance the efficacy of the vaccine, stimulate various immune responses including the enhancement of macrophage activity and induce production of pro-inflammatory cytokines, and can elicit anti-viral responses [35–37]. The addition of hBD3 as the adjuvant showed that the MEVCs had an estimated half-life of at least 30 times longer in both *in vitro* and *in vivo*. The designed MEVCs were extensively evaluated for their probable efficiency to induce immune responses through molecular docking with toll-like receptors (TLRs).

The molecular docking analyses showed that MEVC-B could possibly bind with the cell surface of the TLRs associated with COVID-19 infection, i.e., TLR2 and TLR4 [115, 116]. The binding interactions between the MEVC-B and both TLR molecules were favourable. The interaction between MEVC-B and TLR4 could potentially trigger TLR4 signalling networks, activating immune responses against viral infections [116]. The involvement of residues from the reported binding domains of the TLR4 in the interaction with the MEVC-B showed promising results for the stimulation of the innate immune reaction by the designed MEVC. The high negative value of the binding affinity for the docked complexes generated from both docking tools, ClusPro and HADDOCK indicated that the binding between MEVC-B and the TLR molecules is highly likely to occur. Therefore, the MEVC-B could serve as a potent ligand for both receptors to stimulate the desired immune responses [117]. The results of the *in silico* predictions of the epitopes and evaluations of the designed MEVC indicated that this vaccine candidate can serve as a potential therapeutic strategy for COVID-19 against SARS-CoV-2 variants.

The MD simulation analyses on MEVCB-TLR4-MD-2 complex has shown a promising potential interaction between the vaccine construct, MEVC-B with the TLR4 molecules. The RMSD, RMSF and Rg values which represent the structural stability, flexibility and compactness of the MEVC-B in the protein-protein complex over the 100ns simulation showed that the MEVC-B is stable to be able to interact with TLR4 molecule in the real system. Although the MEVC-B may have shown lower structural stability when in complex with TLR1-TLR2 molecule, we expect that with longer simulation time, the vaccine construct will be able to reach more stable phase and may also have potential to successfully interact with the TLR1-TLR2 molecule as no unfolding of the MEVC-B was observed at different timepoints of the simulation.

As with any computational-based approach, there are limitations to the *in silico* analysis used in this study to design the multi-epitope oral vaccine against SARS-CoV-2 virus. While the use of bioinformatics tools greatly improves the efficiency and accuracy of the vaccine design process, there are still challenges to overcome. One of the limitations of the existing bioinformatics software is the potential inaccuracies of the algorithms used [20], as well as the lack of sufficient information in databases and inherent bias of training datasets. These limitations could potentially compromise the accuracy of molecular screenings and early predictions for vaccine design. Therefore, further experiments such as using immune markers as surrogate [118] to validate the predicted results and improve the accuracy and efficiency of vaccine design is crucial.

To efficiently deliver the designed vaccine, a lactic acid bacteria (LAB), *Lactococcus lactis* (*L*. *lactis*), will be considered in further experimental study. The *L*. *lactis* which has been generally recognised as safe (SAFE) due its long-term use in food products such as cheese and buttermilk [119–121], have been reported as effective microbial cell factories and carriers for therapeutic drug delivery into the gastrointestinal tract (GIT) [120]. The aforementioned advantages of *L*. *lactis* making it a convenient choice as a potential carrier for our vaccine construct [122].

## Conclusion

This immunoinformatic predictions approach for the design of multi-epitope vaccines can provide useful information for researchers to develop an effective and durable therapeutic strategy for diseases management. The quick production of DNA-based multi-epitope vaccine is advantageous for therapeutic prevention, especially in time-sensitive situations like the recent COVID-19 pandemic. The findings in our study suggest that the highly conserved regions of the S protein, particularly the RBD and HR1-HR2 domains, could be promising combined targets for the development of multi-epitope-based vaccines. The MEVC designed in this study could also serve as a potential candidate vaccine against multiple SARS-CoV-2 variants. This study paves the way for the development of an effective and durable therapeutic strategy against COVID-19.

## Supporting information

S1 Table Predicted CTL epitope candidate within receptor-binding domain and heptad repeat domains of the SARS-CoV-2 surface glycoprotein with their antigenicity value, conservancy percentage, binding core and respective binding alleles. (PDF)

S2 Table Predicted HTL epitopes within receptor-binding domain and heptad repeat domains of the SARS-CoV-2 surface glycoprotein with their binding core, respective binding alleles, antigenicity value and conservancy percentage. (PDF)

S3 Table Predicted LBC epitopes within receptor-binding domain and heptad repeat domains of the SARS-CoV-2 surface glycoprotein with their length, region, antigenicity value, conservancy percentage, predicted specific immunoglobulin isotypes and qualitative measurement on specific B-cell assays reported in IEDB resource database. (PDF)

S4 Table Final LBC epitope candidates within receptor-binding domain and heptad repeat domains of the SARS-CoV-2 surface glycoprotein that were reported to have positive binding in B-cell assays in IEDB resource with their specific immunoglobulin isotype, binding core, antigenicity value and conservancy percentage. (PDF)

S5 Table Validation of five models structures of MEVC-B and MEVC-C generated by Robetta web server using ProSA and PROCHECK. (PDF)

S6 Table Model 2 of MEVC-B after refinement with GalaxyRefine. (PDF)

S7 Table Interacting residues within MEVCB-TLR4 complex identified from PDBsum and PRODIGY web servers. (PDF)

S8 Table Interacting residues within MEVCB-TLR1-TLR2 complex identified from PDBsum and PRODIGY web servers. (PDF)

S9 Table Docking results of the individual epitopes and TLR molecules for the top 10 highest binding affinity with the lowest Z-score and HADDOCK score generated by HADDOCK web server. (PDF)
