# Supplementary material for: Engineering receptor-binding domain and heptad repeat domains towards the development of multi-epitopes oral vaccines against SARS-CoV-2 variants
Source: PLoS One. 2024 Aug 15;19(8):e0306111. doi: 10.1371/journal.pone.0306111 (PMC11326571; doi:10.1371/journal.pone.0306111)
Supplement: S1 Table — (PDF) [file pone.0306111.s001.pdf]

S1 Table. Predicted CTL epitope candidate within receptor-binding domain and heptad repeat domains of the SARS-CoV-2 surface glycoprotein with their antigenicity value, conservancy percentage, binding core and respective binding alleles.

No	Epitope	Region	Antigenicity	Conservancy	Alleles
1	^{388}NDLCFTNVY^{396}	RBD	1.6828	98.36%	HLA-B*44:02
2	^{507}PYRVVVLSF^{515}	RBD	1.0281	98.51%	HLA-A*23:01, HLA-A*24:02
3	^{509}RVVVLSFEL^{517}	RBD	1.1918	97.91%	HLA-A*32:01
4	^{349}SVYAWNRKR^{357}	RBD	0.7650	96.86%	HLA-A*03:01, HLA-A*11:01, HLA-A*31:01, HLA-A*33:01, HLA-A*68:01
5	^{342}FNATRFASV^{350}	RBD	0.5609	88.79%	HLA-B*08:01
6	^{1059}GVVFLHVTY^{1067}	HR	1.4104	98.05%	HLA-A*30:02
7	^{1060}VVFLHVTYV^{1068}	HR	1.5122	97.77%	HLA-A*02:01, HLA-A*02:03, HLA-A*02:06, HLA-A*68:02
8	^{1062}FLHVTYVPA^{1070}	HR	1.3346	96.80%	HLA-A*02:03,
9	^{1016}AEIRASANL^{1024}	HR	0.7082	96.52%	HLA-B*40:01, HLA-B*44:02, HLA-B*44:03
10	^{1181}KEIDRLNEV^{1189}	HR	0.5300	95.54%	HLA-B*40:01, HLA-B*44:02, HLA-B*44:03
11	^{1065}VTYVPAQEK^{1073}	HR	0.8132	94.15%	HLA-A*03:01, HLA-A*11:01
12	^{1192}NLNESLIDL^{1200}	HR	0.6827	93.87%	HLA-A*02:01, HLA-A*02:03
13	^{1113}QIITTDNTF^{1121}	HR	0.4253	90.57%	HLA-B*15:01

RBD: Receptor-binding domain; HR: Heptad repeat
