# Supplementary material for: Engineering receptor-binding domain and heptad repeat domains towards the development of multi-epitopes oral vaccines against SARS-CoV-2 variants
Source: PLoS One. 2024 Aug 15;19(8):e0306111. doi: 10.1371/journal.pone.0306111 (PMC11326571; doi:10.1371/journal.pone.0306111)
Supplement: S2 Table — (PDF) [file pone.0306111.s002.pdf]

S2 Table. Predicted HTL epitopes within receptor-binding domain and heptad repeat domains of the SARS-CoV-2 surface glycoprotein with their binding core, respective binding alleles, antigenicity value and conservancy percentage.

No	Epitope	Core	Region	Antigenicity	Conservancy	Alleles
1	^{510}VVVLSFELLHAPATV^{524}	FELLHAPAT	RBD	0.8083	94.32%	DRB1_0101*
2	^{509}RVVVLSFELLHAPAT^{523}	FELLHAPAT	RBD	0.7485	94.17%	DRB1_0101, HLA-DQA10101-DQB10501, HLA-DPA10301-DPB10402
3	^{431}GCVIAWNSNNLDSKV^{445}	IAWNSNNLD	RBD	0.4585	93.57%	DRB1_1501*, DRB3_0202*, DRB1_0401, DRB1_0405, DRB1_1302, HLA-DQA10101-DQB10501
4	^{342}FNATRFASVYAWNRK^{356}	FASVYAWNR	RBD	0.449	86.25%	DRB5_0101*, HLA-DPA10201-DPB10101, HLA-DPA10201-DPB10501, HLA-DPA10201-DPB11401,
5	^{343}NATRFASVYAWNRKR^{357}	FASVYAWNR	RBD	0.4062	86.10%	DRB1_0101, DRB5_0101
6	^{346}RFASVYAWNRKRISN^{360}	YAWNRKRIS	RBD	0.4243	85.95%	DRB1_1101, DRB5_0101
7	^{345}TRFASVYAWNRKRIS^{359}	FASVYAWNR	RBD	0.4963	85.95%	DRB1_0101, DRB5_0101
8	^{1057}PHGVVFLHVTYVPAQ^{1071}	VVFLHVTYV	HR	0.8097	95.68%	DRB1_1201*, HLA-DPA10103-DPB10201*, HLA-DPA10103-DPB10401*, DRB1_0101, DRB1_0701, DRB1_1501, HLA-DQA10101-DQB10501, HLA-DPA10201-DPB10101, HLA-DPA10301-DPB10402, HLA-DPA10201-DPB10501
9	^{1059}GVVFLHVTYVPAQEK^{1073}	FLHVTYVPA	HR	1.1043	93.45%	HLA-DPA10201-DPB10101*, HLA-DPA10103-DPB10201*, HLA-

						DPA10103-DPB10401*, HLA-DPA10301-DPB10402*, HLA-DPA10201-DPB10501*, DRB1_0701, DRB1_0802, HLA-DQA10301-DQB10302
10	^{1062}FLHVTYVPAQEKNFT^{1076}	VTYVPAQEK	HR	1.1908	93.04%	DRB1_0901*, DRB5_0101*, DRB1_0701, HLA-DPA10301-DPB10402, HLA-DPA10201-DPB10501
11	^{1014}RAAEIRASANLAATK^{1028}	IRASANLAA	HR	0.5709	82.45%	DRB1_0401*,DRB4_0101*,DRB1_0101,DRB1_0301,DRB1_0405,DRB1_0701,DRB1_0802,DRB1_0901,DRB1_1101,DRB1_1302,DRB1_1501,DRB3_0202,HLA-DQA10501-DQB10301,HLA-DQA10401-DQB10402,HLA-DQA10102-DQB10602,HLA-DPA10201-DPB11401

*Strong binding allele

RBD: Receptor-binding domain; HR: Heptad repeat
