# Supplementary material for: Engineering receptor-binding domain and heptad repeat domains towards the development of multi-epitopes oral vaccines against SARS-CoV-2 variants
Source: PLoS One. 2024 Aug 15;19(8):e0306111. doi: 10.1371/journal.pone.0306111 (PMC11326571; doi:10.1371/journal.pone.0306111)
Supplement: S3 Table — (PDF) [file pone.0306111.s003.pdf]

S3 Table. Predicted LBC epitopes within receptor-binding domain and heptad repeat domains of the SARS-CoV-2 surface glycoprotein with their length, region, antigenicity value, conservancy percentage, predicted specific immunoglobulin isotypes and qualitative measurement on specific B-cell assays reported in IEDB Resource Database.

No	Epitopes	Length	Region	Antigenicity	Conservancy	Ig Isotypes	B-cell Assays	Qualitative Measurement
1	^{456}FRKSNLKPFERD^{467}	12	RBD	0.7053	97.46%	IgA Epitope	IgG	Positive
2	^{457}RKSNLKPFERDI^{468}	12	RBD	0.6927	95.81%	IgG Epitope	IgG, IgM	Positive
3	^{460}NLKPFERDISTE^{471}	12	RBD	0.5155	95.22%	IgA Epitope	IgG	Positive
4	^{419}ADYNYKLPDDFT^{430}	12	RBD	0.8392	95.07%	IgG Epitope	IgG, IgM	Positive
5	^{457}RKSNLKPFERDISTE^{471}	15	RBD	0.4847	94.47%	IgA Epitope	IgG, IgA	Positive
6	^{437}NSNNLDSKVGGN^{448}	12	RBD	0.6962	92.38%	IgG Epitope	IgA	Positive
7	^{437}NSNNLDSKVGGNYNY^{451}	15	RBD	0.9090	91.33%	IgG Epitope	IgG, IgA	Positive
8	^{411}APGQTGKIADYNYKL^{425}	15	RBD	1.4441	80.57%	IgG Epitope	IgG, IgM, IgA	Positive
9	^{1041}DFCGKGYHLMSF^{1052}	12	HR	0.4493	98.75%	IgG Epitope	IgG	Positive
10	^{1039}RVDFCGKGYHLMS^{1051}	13	HR	0.4949	98.61%	IgG Epitope	NA	NA
11	^{1040}VDFCGKGYHLMSF^{1052}	13	HR	0.4698	98.47%	IgG Epitope	NA	NA
12	^{1106}QRNFYEPQIITT^{1117}	12	HR	0.4354	95.13%	IgA Epitope	IgG, IgM	Positive

13	^{1103}FVTQRNFYEPQII^{1115}	13	HR	0.5425	94.85%	IgA Epitope	NA	NA
14	^{1104}VTQRNFYEPQII^{1115}	12	HR	0.6931	94.85%	IgA Epitope	IgM	Positive
15	^{1103}FVTQRNFYEPQIIT^{1116}	14	HR	0.4614	94.15%	IgA Epitope	NA	NA
16	^{1103}FVTQRNFYEPQIITT^{1117}	15	HR	0.5314	93.04%	IgA Epitope	NA	NA
17	^{1104}VTQRNFYEPQIITT^{1117}	14	HR	0.6522	93.04%	IgA Epitope	NA	NA
18	^{1067}YVPAQEKNFTTA^{1078}	12	HR	0.6277	92.20%	Non-Epitope	IgM, IgG	Positive
19	^{1156}FKNHTSPDVDLG^{1167}	12	HR	1.0976	92.06%	IgG Epitope	NA	NA
20	^{1157}KNHTSPDVDLGD^{1168}	12	HR	1.3336	91.78%	IgG Epitope	NA	NA
21	^{1156}FKNHTSPDVDLGD^{1168}	13	HR	1.0616	91.50%	IgG Epitope	NA	NA
22	^{1157}KNHTSPDVDLGDI^{1169}	13	HR	1.4147	91.50%	IgG Epitope	NA	NA
23	^{1066}TYVPAQEKNFTTA^{1078}	13	HR	0.5531	91.50%	IgG Epitope	NA	NA
24	^{1068}VPAQEKNFTTAPA^{1080}	13	HR	0.6801	90.95%	Non-Epitope	NA	NA
25	^{1067}YVPAQEKNFTTAP^{1079}	13	HR	0.6578	90.95%	Non-Epitope	NA	NA
26	^{1153}DKYFKNHTSPDVDLG^{1167}	15	HR	0.6128	90.81%	IgG Epitope	IgG, IgM, IgA	Positive
27	^{1153}DKYFKNHTSPDVDLGD^{1168}	16	HR	0.6140	90.25%	IgG Epitope	IgG	Positive
28	^{1066}TYVPAQEKNFTTAP^{1079}	14	HR	0.5860	90.25%	IgG Epitope	NA	NA
29	^{1066}TYVPAQEKNFTTAPA^{1080}	15	HR	0.5438	90.11%	IgG Epitope	IgM, IgA	Positive
30	^{1033}VLGQSKRVDFCG^{1044}	12	HR	1.1677	98.89%	IgG Epitope	IgG	Positive

31	^{1155}YFKNHTSPDVDL^{1166}	12	HR	0.4934	93.45%	IgG Epitope	IgG	Positive
32	^{1157}KNHTSPDVDLGDISGI^{1172}	16	HR	1.0583	90.81%	IgG Epitope	IgG	Positive
33	^{1158}NHTSPDVDLGDISGIN^{1173}	16	HR	1.1399	90.53%	IgG Epitope	IgG	Positive
34	^{932}GKIQDSLSSTAS^{943}	12	HR	0.5659	90.39%	IgA Epitope	IgG, IgM	Positive
35	^{931}IGKIQDSLSSTASA^{944}	14	HR	0.5230	90.25%	IgA Epitope	IgG	Positive
36	^{940}STASALGKLQDVVNQN^{955}	16	HR	0.5712	70.47%	IgG Epitope	IgG	Positive

RBD: Receptor-binding domain; HR: Heptad repeat
