# Supplementary material for: Engineering receptor-binding domain and heptad repeat domains towards the development of multi-epitopes oral vaccines against SARS-CoV-2 variants
Source: PLoS One. 2024 Aug 15;19(8):e0306111. doi: 10.1371/journal.pone.0306111 (PMC11326571; doi:10.1371/journal.pone.0306111)
Supplement: S4 Table — (PDF) [file pone.0306111.s004.pdf]

S4 Table. Final LBC epitope candidates within receptor-binding domain and heptad repeat domains of the SARS-CoV-2 surface glycoprotein that were reported to have positive binding in B-cell assays in IEDB Resource with their specific immunoglobulin isotype, binding core, antigenicity value and conservancy percentage.

No	Epitope	Antigenicity	Ig isotype predicted	Conservancy[a]	Assayed antibody from IEDB database
1	^{456}FRKSNLKPFERD^{467}	0.7053	IgA Epitope	97.46%	IgG
2	^{457}RKSNLKPFERDI^{468}	0.6927	IgG Epitope	95.81%	IgG, IgM
3	^{460}NLKPFERDISTE^{471}	0.5155	IgA Epitope	95.22%	IgG
4	^{419}ADYNYKLPDDFT^{430}	0.8392	IgG Epitope	95.07%	IgG, IgM
5	^{457}RKSNLKPFERDISTE^{471}	0.4847	IgA Epitope	94.47%	IgG, IgA
6	^{437}NSNNLDSKVGGN^{448}	0.6962	IgG Epitope	92.38%	IgA
7	^{437}NSNNLDSKVGGNYNY^{451}	0.9090	IgG Epitope	91.33%	IgG, IgA
8	^{1041}DFCGKGYHLMSF^{1052}	0.4493	IgG Epitope	98.75%	IgG
9	^{1106}QRNFYEPQIITT^{1117}	0.4354	IgA Epitope	95.13%	IgG, IgM
10	^{1104}VTQRNFYEPQII^{1115}	0.6931	IgA Epitope	94.85%	IgM
11	^{1153}DKYFKNHTSPDVDLG^{1167}	0.6128	IgG Epitope	90.81%	IgG, IgM, IgA
12	^{1153}DKYFKNHTSPDVDLGD^{1168}	0.6140	IgG Epitope	90.25%	IgG
13	^{1066}TYVPAQEKNFTTAPA^{1080}	0.5438	IgG Epitope	90.11%	IgM, IgA

14	^{1033}VLGQSKRVDFCG^{1044}	1.1677	IgG Epitope	98.89%	IgG
15	^{1155}YFKNHTSPDVDL^{1166}	0.4934	IgG Epitope	93.45%	IgG
16	^{1157}KNHTSPDVDLGDISGI^{1172}	1.0583	IgG Epitope	90.81%	IgG
17	^{1158}NHTSPDVDLGDISGIN^{1173}	1.1399	IgG Epitope	90.53%	IgG
18	^{932}GKIQDSLSSTAS^{943}	0.5659	IgA Epitope	90.39%	IgG, IgM
19	^{931}IGKIQDSLSSTASA^{944}	0.5230	IgA Epitope	90.25%	IgG

[a] Conservancy threshold is 90%
