# Supplementary material for: Engineering receptor-binding domain and heptad repeat domains towards the development of multi-epitopes oral vaccines against SARS-CoV-2 variants
Source: PLoS One. 2024 Aug 15;19(8):e0306111. doi: 10.1371/journal.pone.0306111 (PMC11326571; doi:10.1371/journal.pone.0306111)
Supplement: S5 Table — (PDF) [file pone.0306111.s005.pdf]

S5 Table. Validation of five models structures of MEVC-B and MEVC-C generated by Robetta web server using ProSA and PROCHECK.

**Construct**	**ProSA**		**ERRAT**	**Ramachandran plot**	
	**Z-score**	**Region**		**Most favoured region**	**Disallowed region**
**MEVC-B_1**	-6.44	Within blue region	86.2	81.0	2.3
**MEVC-B _2**	-5.80	Within blue region	87.6	86.8	1.0
**MEVC-B _3**	-6.02	Within blue region	90.6	79.0	2.9
**MEVC-B _4**	-6.09	Within blue region	90.3	78.4	2.1
**MEVC-B _5**	-5.49	Within blue region	90.8	84.1	1.6
**MEVC-C_1**	-6.73	Within blue region	90.1	81.9	1.2
**MEVC-C _2**	-5.52	Within blue region	90.1	84.5	1.0
**MEVC-C _3**	-6.83	Within blue region	93.2	80.2	1.9
**MEVC-C _4**	-6.68	Within blue region	89.3	81.2	2.3
**MEVC-C _5**	-6.20	Within blue region	91.8	80.4	2.5
