# Supplementary material for: Engineering receptor-binding domain and heptad repeat domains towards the development of multi-epitopes oral vaccines against SARS-CoV-2 variants
Source: PLoS One. 2024 Aug 15;19(8):e0306111. doi: 10.1371/journal.pone.0306111 (PMC11326571; doi:10.1371/journal.pone.0306111)
Supplement: S6 Table — (PDF) [file pone.0306111.s006.pdf]

S6 Table. Model 2 of MEVC-B after refinement with GalaxyRefine.

Model	GDT-HA	RMSD	MolProbity	Clash score	Poor rotamers	Rama favoured
Initial	1.0000	0.000	1.496	2.6	0.0	92.8
Model_1	0.9695	0.365	1.885	9.4	0.0	94.2
Model_2	0.9624	0.396	1.871	9.0	0.2	94.2
Model_3	0.9565	0.400	1.989	11.7	0.2	93.9
Model_4	0.9745	0.370	1.903	9.6	0.4	94.1
Model_5	0.9610	0.381	1.873	9.8	0.2	94.8
