# Supplementary material for: Engineering receptor-binding domain and heptad repeat domains towards the development of multi-epitopes oral vaccines against SARS-CoV-2 variants
Source: PLoS One. 2024 Aug 15;19(8):e0306111. doi: 10.1371/journal.pone.0306111 (PMC11326571; doi:10.1371/journal.pone.0306111)
Supplement: S7 Table — (PDF) [file pone.0306111.s007.pdf]

S7 Table. Interacting residues within MEVCB-TLR4 complex identified from PDBsum and PRODIGY web servers.

No.	PDBsum				PRODIGY			
	**TLR4-A**	**MEVC-B**	**TLR4-B**	**MEVC-B**	**TLR4-A**	**MEVC-B**	**TLR4-B**	**MEVC-B**
1	Arg382	Gln418	Arg460	Ala442	Ser381	Gln418	Arg460	Ala442
2	Tyr403	Lys420	Ala462	Tyr443	Arg382	Lys420	Val461	Tyr443
3	Asp405	Pro422	Gln484	Cys447	Lys402	Gly421	Ala462	Asn444
4	Ser407	Ala465	Glu485	Phe448	Tyr403	Pro422	Phe463	Cys447
5	Phe408	Ala466	Phe487	Val451	Asp405	Gly423	Asn464	Phe448
6	Glu425	Arg468	Asp490	Tyr452	Ser407	Leu462	Phe483	Val451
7	His426	Val469	Gln510	Tyr455	Phe408	Phe464	Gln484	Tyr452
8	Asp428	Leu472	Leu511	Arg458	Glu425	Ala465	Glu485	Tyr455
9	Gln430	Trp484	Ser512	Leu462	His426	Ala466	Phe487	Arg458
10	His431	Arg486	Pro513	Ser463	Asp428	Tyr467	Leu488	Val459
11	Ile450	Arg488	Thr514	Tyr503	Gln430	Arg468	Pro489	Leu462
12	Tyr451	Tyr491	Asp536	Phe506	His431	Val469	Asp490	Ser463
13	Val475	Phe495	Phe538	Leu507	Ile450	Val470	Thr493	Tyr503
14	Gln505	Tyr503	Lys561	Val509	Tyr451	Leu472	Gln510	Phe506
15	Ser528		Gln562	Thr510	Asp453	Ser473	Leu511	Leu507
16	Ser552		Glu563	Ala513	His456	Phe474	Ser512	Val509
17	Leu553			Thr517	Glu474	Ala477	Pro513	Thr510
18	Asn575			Lys524	Val475	Ser480	Thr514	Tyr511
19	Gln578				Lys477	Val481	Asp536	Ala513
20	Glu603				Phe500	Trp484	Thr537	Ala514
21	Glu605				Asp502	Arg486	Phe538	Val516
22	Arg606				Gln505	Arg488	Pro539	Thr517
23	Glu608				Val524	Tyr491	Lys560	Ala521
24	Asp614				Asn526	Gly492	Lys561	Lys524
25	Lys615				Ser528	Phe495	Gln562	
26	Met618				His529	Leu496	Glu563	
27					Asp550	Thr499	Leu564	
28					Ser552	Tyr503	Gln565	
29					Leu553			
30					Asn575			
31					Thr577			
32					Gln578			
33					Glu603			
34					Glu605			
35					Arg606			
36					Met607			
37					Glu608			
38					Ser613			
39					Asp614			

40					Lys615			
41					Gln616			
42					Met618			
