# Supplementary material for: Engineering receptor-binding domain and heptad repeat domains towards the development of multi-epitopes oral vaccines against SARS-CoV-2 variants
Source: PLoS One. 2024 Aug 15;19(8):e0306111. doi: 10.1371/journal.pone.0306111 (PMC11326571; doi:10.1371/journal.pone.0306111)
Supplement: S8 Table — (PDF) [file pone.0306111.s008.pdf]

S8 Table. Interacting residues within MEVCB-TLR1-TLR2 complex identified from PDBsum and PRODIGY web servers.

No.	PDBsum		PRODIGY	
	TLR1	MEVC-B	TLR1	MEVC-B
1	Lys33	Pro384	Lys33	Asn374
2	His38	Tyr457	Asn34	Pro384
3	Gln54	Val460	Gly35	Gly423
4	Asn55	Phe464	Ile37	Pro424
5	Tyr56	Tyr467	His38	Tyr457
6	His78	Arg468	Ser53	Val460
7	Asn79	Trp484	Gln54	Val461
8	Arg80	Arg486	Asn55	Ser463
9	His102	Lys487	Tyr56	Phe464
10	Lys104	Arg488	His78	Tyr467
11	Val106	Tyr491	Asn79	Arg468
12	Phe123	Phe495	Arg80	Val471
13	Asn124	Val498	Gln82	Trp484
14	Ala125	Ala502	His102	Arg486
15	Val170	Tyr503	Asn103	Lys487
16	Glu173	Val504	Lys104	Arg488
17	Val195	Val505	Val106	Tyr491
18	Thr198	Phe506	Phe123	Phe495
19	Asn199	His508	Asn124	Val498
20	Asn220	Val509	Ala125	Thr499
21	Asn252	Tyr511	Val170	Ala502
22	Asn253	Val512	Leu171	Tyr503
23	Ser279	Tyr515	Gly172	Val504
24	Asn280		Glu173	Val505
25	Ser303		Thr174	Phe506
26	His305		His193	Leu507
27	Gln306		Val195	His508
28	Thr332		Phe196	Val509
29	His352		Thr198	Tyr511
30	Thr376		Asn199	Val512
31	Gln380		Ser219	Ala513
32	Gln402		Asn220	Tyr515
33	Asp404		Thr250	Val516
34	Ser406		Asn252	
35	Gln407		Asn253	
36	Ser427		Ser279	
37	Asn429		Asn280	
38	Ser431		Leu302	
39	His453		Ser303	
40			His305	

41			Gln306	
42			Asn330	
43			Thr332	
44			His352	
45			Asp354	
46			Thr376	
47			Ile378	
48			Gln380	
49			Gln402	
50			Leu403	
51			Asp404	
52			Ile405	
53			Ser406	
54			Gln407	
55			Ser427	
56			Leu428	
57			Asn429	
58			Ser431	
59			Ser432	
60			Val449	
61			Asp451	
62			His453	
