# Supplementary material for: Engineering receptor-binding domain and heptad repeat domains towards the development of multi-epitopes oral vaccines against SARS-CoV-2 variants
Source: PLoS One. 2024 Aug 15;19(8):e0306111. doi: 10.1371/journal.pone.0306111 (PMC11326571; doi:10.1371/journal.pone.0306111)
Supplement: S9 Table — (PDF) [file pone.0306111.s009.pdf]

S9 Table. Docking results of the individual epitopes and TLR molecules for the top 10 highest binding affinity with the lowest Z-score and HADDOCK score generated by HADDOCK web server.

Complex	Epitope Number *	Epitope	HADDOCK Score	Z-Score	Free binding energy (kcal mol^{-1})
MEVCB-TLR4-MD-2	27	^{509}RVVVLSFEL^{517}	-47.0 +/- 10.1	-2.0	-15.0
	19	^{931}IGKIQDSLSSTASA^{944}	-45.5 +/- 24.9	-2.6	-14.2
	22	^{1057}PHGVVFLHVTYVPAQ^{1071}	-66.9 +/- 8.9	-1.4	-13.5
	21	^{431}GCVIAWNSNNLDSKV^{445}	-47.0 +/- 15.8	-1.4	-13.5
	15	^{1158}NHTSPDVDLGDISGIN^{1173}	-48.6 +/- 6.5	-2.1	-13.1
	24	^{1062}FLHVTYVPAQEKNFT^{1076}	-66.9 +/- 8.9	-1.4	-13.0
	20	^{509}RVVVLSFELLHAPAT^{523}	-53.5 +/- 12.2	-2.4	-12.9
	12	^{1153}DKYFKNHTSPDVDLGD^{1168}	-61.4 +/- 3.4	-1.5	-12.9
	14	^{1157}KNHTSPDVDLGDISGI^{1172}	-35.1 +/- 16.7	-1.5	-12.6
	1	^{456}FRKSNLKPFERD^{467}	-68.5 +/- 14.5	-2.7	-12.5
MEVCB-TLR1-TLR2	19	^{931}IGKIQDSLSSTASA^{944}	-58.5 +/- 6.3	-2.1	-13.9
	5	^{437}NSNNLDSKVGGNYNY^{451}	-85.4 +/- 2.6	-1.8	-12.4
	10	^{1104}VTQRNFYEPQII^{1115}	-87.7 +/- 1.0	-2.1	-12.0
	2	^{419}ADYNYKLPDDFT^{430}	-86.2 +/- 1.8	-1.9	-11.7
	12	^{1153}DKYFKNHTSPDVDLGD^{1168}	-93.5 +/- 5.3	-1.4	-11.4
	21	^{431}GCVIAWNSNNLDSKV^{445}	-84.5 +/- 2.5	-2.2	-11.2
	14	^{1157}KNHTSPDVDLGDISGI^{1172}	-59.6 +/- 4.0	-1.6	-11.2
	26	^{507}PYRVVVLSF^{515}	-110.4 +/- 2.1	-2.1	-11.1
	15	^{1158}NHTSPDVDLGDISGIN^{1173}	-70.6 +/- 2.7	-2.1	-11.0
	22	^{1057}PHGVVFLHVTYVPAQ^{1071}	-109.3 +/- 5.8	-2	-11.0

*Based on arrangement in the MEVC-B in Fig 3.
